# Prmt6 Deficiency or Inhibition Restores Microglial Homeostasis and Promotes Scar‐Limited Repair in Adult Spinal Cord Injury

**DOI:** 10.1002/advs.75325

**Published:** 2026-04-17

**Authors:** Weilin Peng, Zhengqiang Wu, Yu Xiong, Zhongya Gao, Yishan Liu, Ziyi Wang, Haibin Wang, Chaofeng Han, Wenxiang Chu, Xuhua Lu

**Affiliations:** ^1^ Department of Orthopaedics Changzheng Hospital Naval Medical University Shanghai China; ^2^ Department of Histology and Embryology National Key Laboratory of Immunity and Inflammation Naval Medical University Shanghai China

**Keywords:** fatty acid oxidation, homeostasis, microglia, PGC‐1α, spinal cord injury

## Abstract

Neonatal mice achieve scar‐free healing after spinal cord injury (SCI) by restoring microglial homeostasis, unlike adults, where persistent microglial dyshomeostasis drives scar expansion through mechanisms that remain elusive. Using RNA sequencing, we identified protein arginine methyltransferase 6 (PRMT6) as a key regulator of this disparity, upregulated in activated microglia at adult SCI lesions but maintained at low levels in neonatal microglia after injury. In adult mice, *Prmt6* deficiency restored microglial homeostasis, evidenced by increased P2Y12/TMEM119 and reduced CD68, while reducing scar formation and enhancing axonal regrowth and motor recovery. Microglia‐specific *Prmt6* knockdown or PRMT6 inhibitor administration recapitulated these effects. Mechanistically, PRMT6 deposits H3R2me2a at the *Ppargc1a* promoter to repress peroxisome proliferator‐activated receptor‐γ coactivator‐1α（PGC‐1α）, thereby inhibiting fatty acid oxidation (FAO) and disrupting microglial homeostasis. Loss of *Prmt6* alleviates this epigenetic repression, restoring FAO and microglial homeostasis. These findings establish PRMT6 as a novel epigenetic regulator linking microglial dyshomeostasis and metabolic dysfunction to maladaptive scar formation in adult SCI, highlighting PRMT6 inhibition as a promising therapeutic strategy to reprogram microglial metabolism and promote neural repair.

## Graphical abstract

After spinal cord injury, adult microglia remain persistently activated with chronic PRMT6 (protein arginine methyltransferase 6) upregulation. *Prmt6* deficiency or inhibition reestablishes microglial homeostasis and promotes a scar‐limited repairment, enhancing axonal regrowth. Mechanistically, PRMT6 promotes asymmetric dimethylation of H3R2 at the *Ppargc1a* promoter and disrupts PGC‐1α‐regulated fatty acid oxidation, which underpins microglial homeostatic programming.

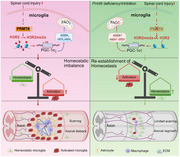

## 1 Introduction

Traumatic spinal cord injury (SCI) is a devastating neurotrauma, leading to premature mortality and long‐term disability [1, 2]. The immune response plays a central role in the complex pathology of SCI [3, 4], in which the mechanism remains elusive.

Microglia, the primary myeloid cells in the central nervous system (CNS), function in two fundamental states: homeostasis and activation. Under physiological conditions, homeostatic microglia constantly surveil the CNS through P2Y12 (purinergic receptor P2Y, G‐protein coupled, 12)‐dependent interactions with neurons [5]. Upon CNS injury or inflammation, microglia rapidly transit to an active state, characterized by downregulation of homeostasis markers such as TMEM119 (transmembrane protein 119) and P2Y12 and upregulation of activation markers such as CD68 [6, 7], contributing to the inflammatory response after SCI [8].

Recent studies highlighted the therapeutic potential of restoring microglial homeostasis after CNS trauma and disorders. In acute brain injury model, inhibition of the P2Y12 receptor exacerbates neuronal damage [5]. Similarly, in Alzheimer's disease (AD), restoring homeostatic microglia signature accelerates the clearance of Aβ and prevented neuronal loss [9, 10]. In multiple sclerosis patients, remyelination during recovery is associated with restored microglial homeostasis [11]. Notably, Li et al. [6] have shown that in neonatal mice, microglia undergo a transient activation and reestablish a homeostatic state within the first week after SCI, promoting scar‐free healing and axon regeneration. In contrast, following adult SCI, microglia fail to re‐establish homeostasis, leading to a reparative process characterized by scar formation [12]. However, the mechanisms underlying the successful restoration of microglial homeostasis in neonates, and the failure to achieve this in adult SCI, remain poorly understood.

To restore microglial homeostasis, it is essential to investigate the mechanisms regulating microglial responses to SCI. Recent studies have increasingly highlighted the role of cellular metabolism in shaping innate immune responses. After traumatic brain injury (TBI), 4‐octyl itaconate (OI) supplementation rescues the downregulated mitochondria OXPHOS and fatty acid oxidation (FAO) in microglia, attenuating neuroinflammation [13]. In AD, inhibiting hexokinase 2 (HK2) promotes fatty acid metabolism and increases ATP levels, facilitating β‐amyloid clearance [14]. Additionally, inhibiting fatty acid oxidation (FAO) in macrophages reduces anti‐inflammatory responses while promoting pro‐inflammatory cytokine production [15]. Furthermore, pyruvate kinase isozyme type M2 (PKM2) inhibition attenuates microglial activation by reducing the permissive histone lactylation at glycolytic gene promoters [16], underscoring the pivotal role of epigenetic modifications in microglial activation. Microglia undergo dynamic chromatin landscape shifts during development [17, 18], indicating that epigenetic factors may contribute to differential SCI responses between neonatal and adult microglia. During early brain development, ARID1A deficiency disrupts microglial homeostasis by altering chromatin modifications, leading to disruption of neural development and autism‐like behaviors [19]. Together, these studies suggest that metabolic and epigenetic regulation are crucial for modulating microglial activation and homeostasis.

PRMT6 (protein arginine methyltransferase 6), a member of the PRMT family, has been implicated in various pathological conditions, including tumorigenesis, antiviral immunity, and neurodegeneration [20, 21, 22, 23]. PRMT6 asymmetrically dimethylates arginine residues on histones, transcription factors, and coregulators [24, 25]. Our previous study demonstrated the epigenetic role of PRMT6 in mediating cellular metabolism during RANKL‐induced osteoclastogenesis [26]. However, its role in mediating microglial functional phenotype in the context of SCI remains unclear.

In this study, RNA‐seq analysis and subsequent immunofluorescence revealed a significant upregulation of PRMT6 in dyshomeostatic microglia at the lesion site following adult SCI. In contrast, neonatal microglia maintained relatively low PRMT6 expression both pre‐ and post‐injury. Using genetic *Prmt6* deficiency, *Cx3cr1*‐miRsh*Prmt6* knockdown of adeno‐associated virus, and pharmacological inhibition of PRMT6, microglial restored homeostasis, resulting in a scar‐limited repair and axonal regrowth. Mechanistically, PRMT6 catalyzed asymmetric dimethylation of the H3R2 residue at the *Ppargc1a* promoter and suppressed PGC‐1α‐mediated FAO, thereby disrupting microglial homeostasis. These findings reveal the epigenetic regulation of microglial immunometabolism after SCI and suggest that targeting PRMT6 could be a promising therapeutic strategy for SCI.

## 2 Results

### 2.1 PRMT6 Upregulation in Adult, but not Neonatal, Microglia

To pinpoint regulators of sustained microglial activation after adult SCI, we performed bulk RNA‐seq on spinal cord tissue at 0, 3, 7, and 14 dpi (Figure S1A). Principal component analysis (PCA) revealed clear separation of samples across time points (Figure S1B). Time‐series clustering identified three modules with genes persistently upregulated (profiles 17–19) (Figure S1C). Gene Ontology (GO) enrichment of the persistently induced modules highlighted processes linked to microglial activation, including “cellular response to stress,” “regulation of cellular metabolic process,” [27] “Regulation of organelle organization” [28, 29] and “Regulation of transcription by RNA polymerase II” among others [29], (Figure S1D), yielding 25 candidate genes by Venn analysis (Figure S1E). To investigate regulators in microglial homeostasis imbalance precisely, RNA‐seq was performed on primary microglia treated with lipopolysaccharide (LPS), a well‐established in vitro model for microglial activation (Figure S1F) [30, 31, 32]. PCA showed clear segregation between conditions (Figure S1G). Applying the same GO filters identified 17 LPS‐responsive genes (Figure S1H,I). Intersection of the spinal cord‐ and microglia‐derived candidate sets yielded five shared genes, including *Prmt6*, *Nod2*, *Il1b*, *Il1a*, and *Tnf*, all upregulated in both injured spinal cords and activated microglia (Figure S1J–L). Given the well‐established proinflammatory functions of IL‐1β, IL‐1α, TNF‐α [33, 34]and NOD2 [35], we focused on *Prmt6* as a previously unappreciated regulator of microglial activation in SCI. Transcript per million (TPM) from both datasets confirmed robust induction of *Prmt6* in vivo and in vitro (Figure S1M,N).

**FIGURE 1:**
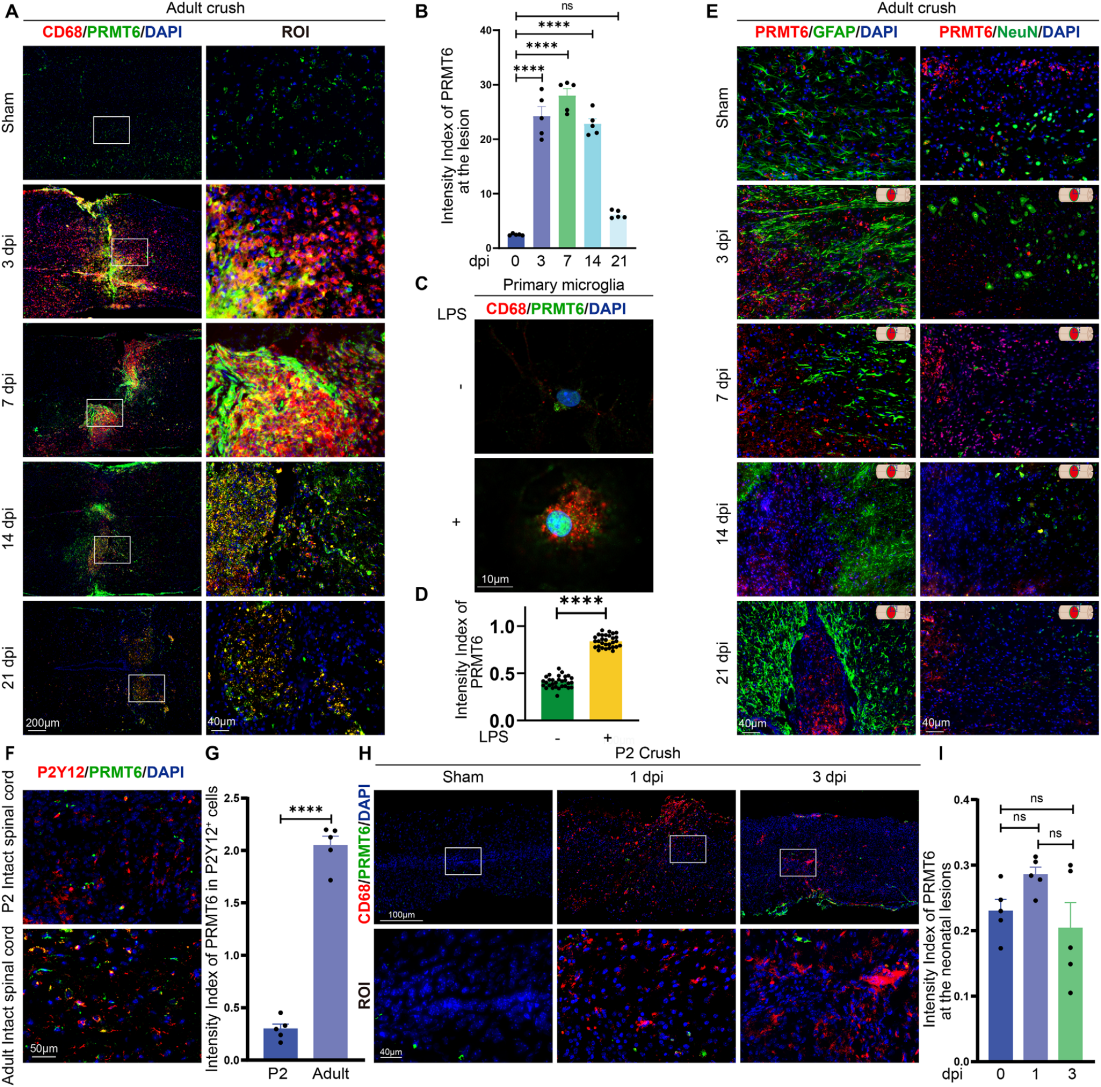
PRMT6 upregulation in adult lesions and activated primary microglia, but not in neonatal lesions. (A,B) Representative images of co‐staining for PRMT6 and CD68 (A) and quantitative analysis of PRMT6 intensity (B) in adult mice from sham and 3, 7, 14, 21 dpi groups (*n* = 5 animals per group). The boxed border regions were magnified. (C,D) Representative images of co‐staining for PRMT6 and CD68 in primary microglia (C) and quantitative analysis of PRMT6 intensity (D) in cells treated with or without LPS (*n* = 30 cells per group). (E) Co‐staining of PRMT6 and GFAP and NeuN in sham, 3, 7, 14, and 21 dpi groups, showing no significant upregulation of PRMT6 in astrocytes or neurons at lesion site. F, G) Representative images of co‐staining for PRMT6 and P2Y12 (F) and quantification of PRMT6 intensity (G) in microglia in intact adult and P2 (postnatal day 2) spinal cord (*n* = 5 animals per group). H, I) Images of immunostaining for PRMT6 and CD68 and (H) quantitative analysis of PRMT6 intensity in P2 mice (I) under uninjured, 1 dpi, and 3 dpi conditions (*n* = 5 animals per group). Values are mean ± SEM. *p < 0.05, **p < 0.01, ***p < 0.001, ****p < 0.0001.

In vivo, PRMT6 was upregulated in activated microglia at 3, 7, and 14 dpi, as shown by its co‐localization with CD68 (Figure 1A,B) and IBA1 (Figure S2) [36]. Consistently, LPS‐activated primary microglia also showed increased PRMT6 expression in vitro (Figure 1C,D). To assess whether PRMT6 induction after SCI occurred in other cell types, we co‐stained PRMT6 with GFAP (glial fibrillary acidic protein, astrocytes) and NeuN (neuron‐specific nuclear protein, neurons). PRMT6 signals remained low in GFAP^+^ astrocytes and NeuN^+^ neurons before injury and at 3, 7, 14, and 21 dpi (Figure 1E), indicating that PRMT6 upregulation in the injured spinal cord is mainly attributable to activated microglia.

To investigate whether PRMT6 contributes to the distinct microglial responses to SCI in adult versus neonatal mice, we examined its expression in neonates. In intact spinal cord, postnatal day‐2 (P2) mice showed relatively lower PRMT6 levels in microglia compared to adults (Figure 1F,G). During neonatal microglial activation after SCI, PRMT6 expression remained low and did not increase in CD68^+^ cells at lesion sites at 1 and 3 dpi (Figure 1H,I). Thus, unlike the persistent induction of PRMT6 in adult microglia after crush injury, neonatal microglia did not upregulate PRMT6, suggesting that differential PRMT6 expression may contribute to the distinct microglial phenotypes following SCI in adults versus neonates.

### 2.2 *Prmt6* Deficiency Re‐Established Microglial Homeostasis and Promoted Scar‐Limited Repair in Adult Mice

**FIGURE 2:**
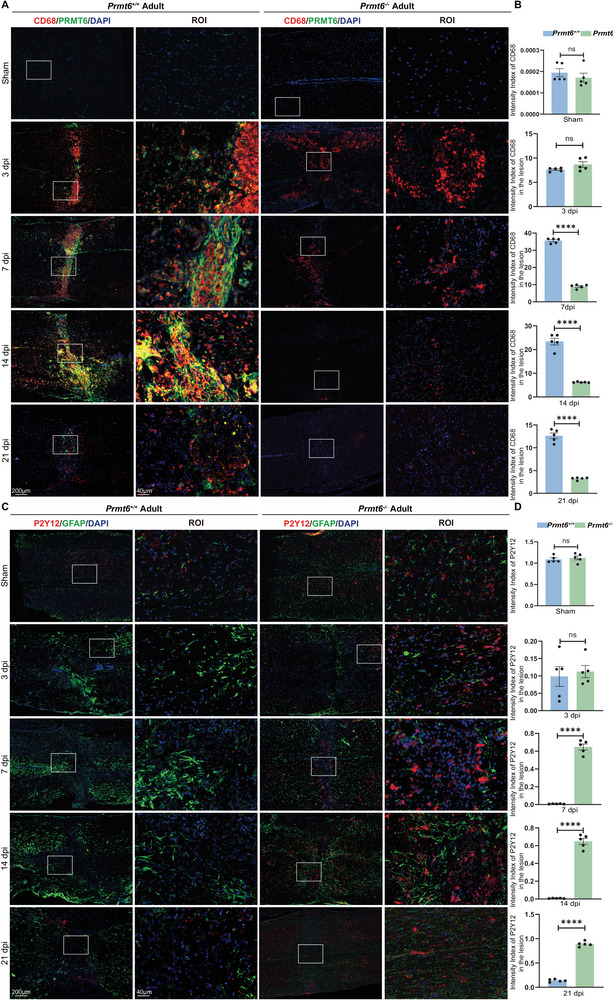
*Prmt6* deficiency re‐established microglial homeostasis after adult SCI. A) Representative images of spinal cord lesions from *Prmt6* ^+/+^ and *Prmt6* ^−/−^ mice at sham, 3, 7, 14, and 21 dpi, stained for CD68 (red) and PRMT6 (green). Nuclei are counterstained with DAPI (blue). Boxed square was magnified. B) Quantification of CD68 immunoreactive intensity in the lesion sites of *Prmt6* ^+/+^ and *Prmt6* ^−/−^ mice of sham, 3, 7, 14, and 21 dpi groups (*n* = 5 animals per group). C) Representative images of spinal cord lesions from *Prmt6* ^+/+^ and *Prmt6* ^−/−^ mice at different time points post‐SCI, stained with antibodies against P2Y12 (red) and GFAP (green), showcasing the re‐establishment of microglial homeostasis in *Prmt6* ^−/−^ mice compared to *Prmt6* ^+/+^ mice. Nuclei were counterstained with DAPI (blue). Boxed square was magnified. D) Quantification of P2Y12 immunoreactive intensity at lesion sites in *Prmt6* ^+/+^ and *Prmt6* ^−/−^ mice at sham, 3, 7, 14, and 21 dpi (*n* = 5 animals per group). Values are plotted as means ± SEM. ****p < 0.0001.

To investigate the role of PRMT6 in regulating microglial activation in adult mice after SCI, we performed immunostaining for the microglial activation marker CD68 and the well‐established homeostatic markers P2Y12 and TMEM119 [5, 6]. In the sham group, *Prmt6* ^+/+^ and *Prmt6* ^−/−^ mice showed similar CD68 and P2Y12 immunoreactivity in the T10 segment (Figure 2A–D), and PRMT6 staining was absent in *Prmt6* ^−/−^ mice, confirming efficient knockout. At 3 dpi, both genotypes exhibited a similar acute activation profile, with increased CD68 and reduced P2Y12/TMEM119 fluorescence intensity at the lesion site (Figure 2A–D; Figure S3A,B). From 7 to 21 dpi, however, CD68 intensity remained high in *Prmt6* ^+/+^ mice but was markedly reduced in *Prmt6* ^−/−^ mice, whereas the microglial homeostatic markers P2Y12 and TMEM119 were significantly higher in *Prmt6* ^−/−^ lesions than in *Prmt6* ^+/+^ controls (Figure 2A–D; Figure S3A,B). Spatially, P2Y12^+^ and TMEM119^+^ microglia in *Prmt6* ^−/−^ mice displayed a ramified morphology and were distributed both around and within the core of the lesion (Figure 2C,D; Figure S3A,B).

**FIGURE 3:**
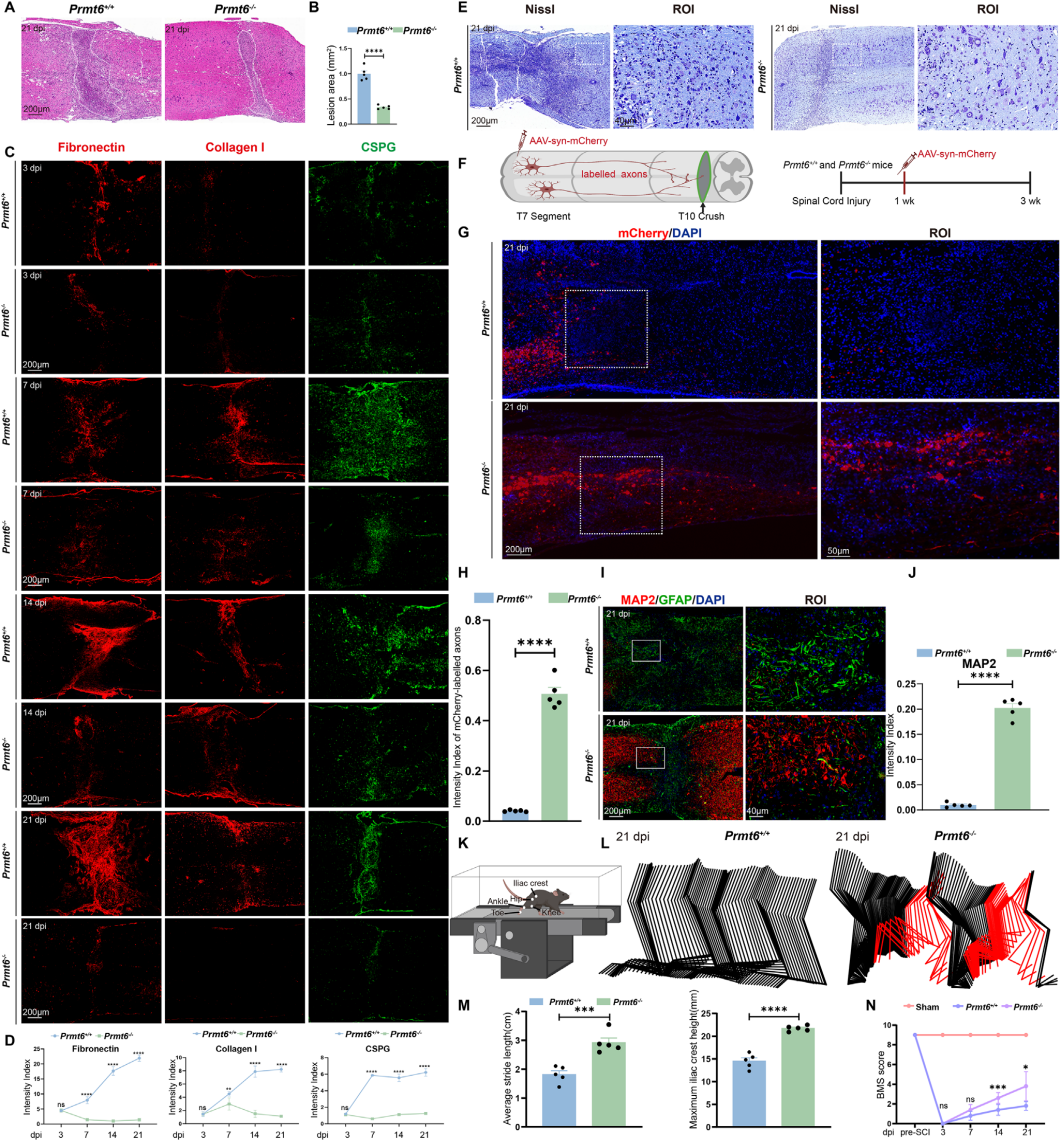
*Prmt6* deficiency led to scar‐limited repair, permitting axonal regeneration and functional recovery. (A,B) Representative HE staining of spinal cord (A) and quantification of lesion area (B) in *Prmt6* ^+/+^ and *Prmt6* ^−/−^ mice at 21 dpi (*n* = 5 animals per group). (C) Representative images of spinal cord of sham, 3, 7, 14, and 21 dpi groups from *Prmt6* ^+/+^ and *Prmt6* ^−/−^ mice, stained with antibodies against Fibronectin, Collagen I, and CSPG. (D) Quantification of immunoreactive Fibronectin, Collagen I, and CSPG intensity at lesion sites in C (*n* = 5 animals per group). (E) Representative images of Nissl staining of spinal cord of *Prmt6* ^+/+^ and *Prmt6* ^−/−^ mice at 21 dpi, showing preserved neuronal morphology in *Prmt6* ^−/−^ mice compared with *Prmt6* ^+/+^ controls. (F) Schematic of the experimental design for tract‐tracing of propriospinal axons with ssAAV‐hSyn‐mCherry. Created in BioRender. Weilin, Peng. (2026) https://BioRender.com/tl2mwmh. (G,H) mCherry‐labelled axons in spinal cord at 21 dpi (G) and quantification of mCherry intensity within the lesion region (H) in *Prmt6* ^+/+^ and *Prmt6* ^−/−^ mice (*n* = 5 animals per group). Boxed regions in the lesion core are shown at higher magnification. (I,J) Co‐staining of GFAP and MAP2 (I) and quantification of MAP2 (J) in lesion areas of *Prmt6* ^+/+^ and *Prmt6* ^−/−^ mice at 21 dpi. The boxed border regions were magnified. (K,L) Schematic illustration of functional assessment (K) and representative stick diagrams of hindlimb movements (L) of *Prmt6* ^+/+^ and *Prmt6* ^−/−^ mice at 21 dpi. Created in BioRender. Weilin, Peng. (2026) https://BioRender.com/tl2mwmh. (M,N) Average stride length and maximum iliac crest height at 21 dpi (M) and BMS scores over 21 days (N) in *Prmt6* ^+/+^ and *Prmt6* ^−/−^ mice (*n* = 5 animals per group). Values are mean ± SEM. *p < 0.05, **p < 0.01, ***p < 0.001, ****p < 0.0001.

Formation of inhibitory scar tissue, a barrier to axonal regeneration, is closely linked to microglial dyshomeostasis after SCI [6, 37]. In line with the restored microglial homeostasis in *Prmt6* ^−/−^ mice, HE staining at 21 dpi revealed a smaller lesion area compared with *Prmt6* ^+/+^ controls (Figure 3A,B). Consistently, immunostaining for scar‐associated ECM components (fibronectin, collagen I, and CSPG) showed reduced deposition in *Prmt6* ^−/−^ spinal cord lesions at 7, 14, and 21 dpi (Figure 3C,D), indicating a more restricted inhibitory scar.

To assess neural repair, we performed Nissl staining. Perilesional neurons in *Prmt6* ^−/−^ mice showed relatively intact morphology with well‐preserved Nissl bodies, whereas neurons surrounding the lesion in *Prmt6* ^+/+^ mice displayed irregular shapes and blurred contours (Figure 3E). For axonal regeneration, propriospinal axons were traced with ssAAV‐Syn‐mCherry injected 7 days after SCI (Figure 3F). By 21 dpi, *Prmt6* ^−/−^ mice showed higher densities of mCherry‐labelled axons at and caudal to the lesion compared with *Prmt6* ^+/+^ counterparts (Figure 3G,H). Consistently, NF‐H immunostaining revealed increased axonal fibers crossing the lesion in *Prmt6* ^−/−^ mice at 7, 14, and 21 dpi (Figure S4), and MAP2 staining demonstrated more neuronal structures within the lesion area at 21 dpi (Figure 3I,J).

Finally, we evaluated locomotor recovery in *Prmt6* ^+/+^ and *Prmt6* ^−/−^ mice after SCI [38]. Hindlimb gait analysis were recorded on a treadmill by marking the iliac crest, hip, knee, ankle, and toe to track movement trajectories (Figure 3K). At 21 dpi, *Prmt6* ^+/+^ mice showed persistent hindlimb ankle flexion with abnormal gait and poor weight support (Figure 3L). By contrast, *Prmt6* ^−/−^ mice exhibited greater ankle dorsiflexion, increased stride length, higher iliac crest position, and elevated BMS scores (Figure 3M,N), indicating improved motor function recovery.

In summary, our data identified PRMT6 as a central driver of microglial homeostatic imbalance during SCI. *Prmt6* deficiency restored a homeostatic microglial phenotype, limited inhibitory scar formation, and thereby facilitated axonal regeneration and functional recovery after adult SCI, highlighting PRMT6 as a promising therapeutic target.

### 2.3 *Prmt6* Deficiency in Microglia, not in Blood‐Borne Cells, was Necessary for the Re‐Establishment of Microglial Homeostasis

Given that PRMT6 was upregulated in CD68^+^ cells (microglia and blood‐borne macrophages), to dissect the relative contribution of PRMT6 in resident microglia versus blood‐borne macrophages after SCI, we selectively deleted *Prmt6* in microglia or in infiltrating macrophages.

**FIGURE 4:**
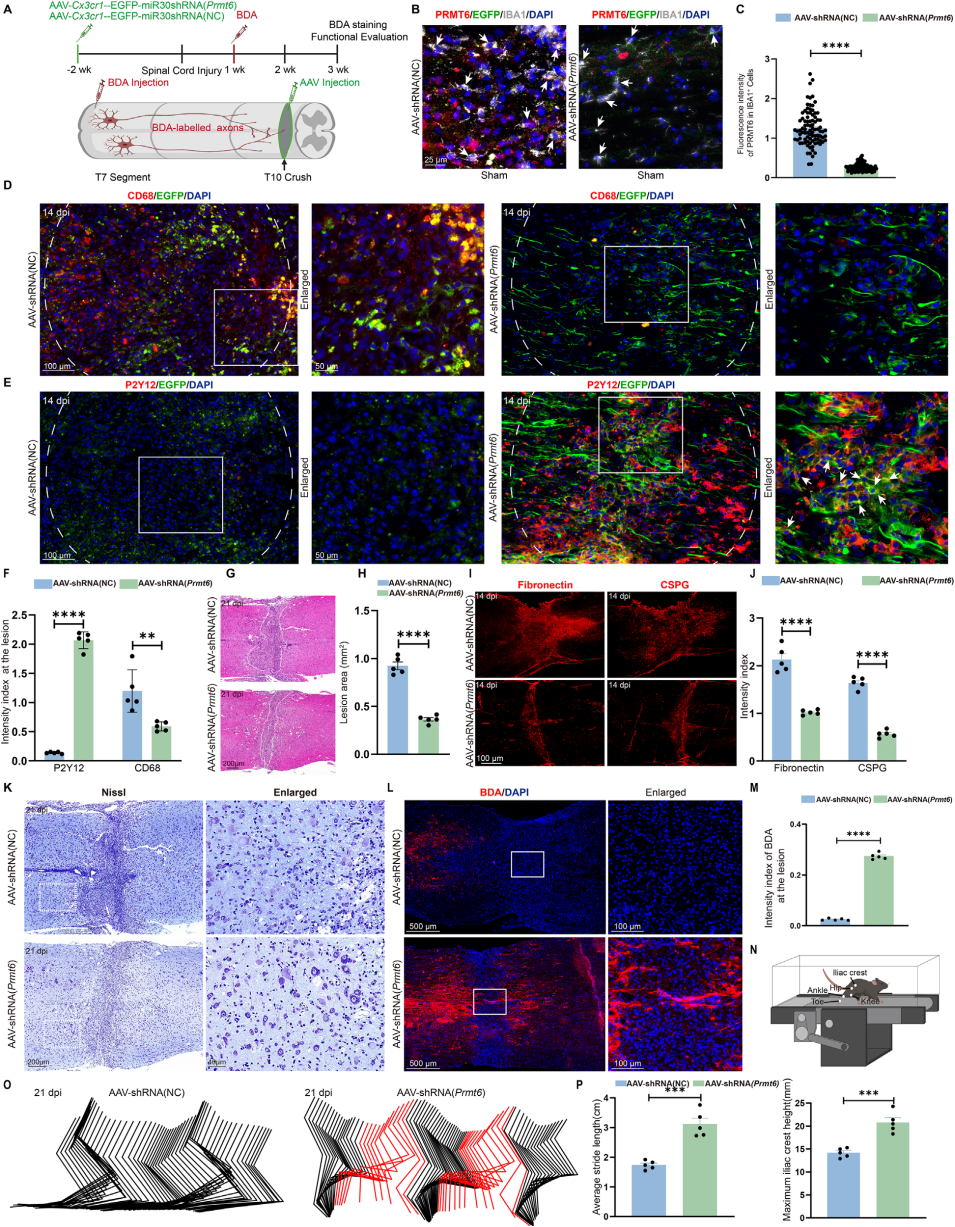
Microglia‐specific *Prmt6* knockdown restored microglial homeostasis and promoted tissue repair. (A) Schematic of the experimental timeline and injection site for AAV‐*Cx3cr1*‐EGFP‐miR30shRNA(*Prmt6*) or AAV‐*Cx3cr1*‐EGFP‐miR30shRNA(NC). Created in BioRender. Weilin, Peng. (2026) https://BioRender.com/tl2mwmh. (B,C) Representative images of co‐staining for PRMT6, EGFP, and IBA1 in intact spinal cord injected with control or *Prmt6* shRNA AAV (B) and quantification of PRMT6 expression in IBA1^+^ microglia (C) (*n* = 30 cells per animal, 3 animals per group). White arrows indicate microglia. (D) Representative images of CD68 and EGFP co‐staining at 14 dpi in spinal cords infected with control or *Prmt6* shRNA AAV. Boxed regions are shown at higher magnification; dotted lines mark lesion borders. (E) Representative images of P2Y12 and EGFP co‐staining at 14 dpi in spinal cord infected with control or *Prmt6* shRNA AAV. Boxed regions are shown at higher magnification; dotted lines mark lesion borders. (F) Quantification of CD68 and P2Y12 intensity at lesion sites in D and E (*n* = 5 animals per group). (G,H) Representative HE staining of spinal cord (G) and quantification of lesion area (H) at 21 dpi in control and *Prmt6* shRNA AAV groups (*n* = 5 animals per group). (I,J) Representative images of fibronectin and CSPG staining at 14 dpi in control and *Prmt6* shRNA AAV‐infected spinal cord (I) and quantification of fibronectin and CSPG intensity (J) at lesion sites (*n* = 5 animals per group). (K) Representative Nissl staining of spinal cord at 21 dpi in control and *Prmt6* shRNA AAV groups. (L,M) Representative images of BDA‐labelled axons at 21 dpi (L) and quantification of BDA intensity within lesion sites (M) in control and *Prmt6* shRNA AAV‐treated mice (*n* = 5 animals per group). Boxed regions are shown at higher magnification. (N,O) Schematic of gait assessment (N) and representative stick diagrams of hindlimb movements (O) at 21 dpi in control and *Prmt6* shRNA AAV‐treated mice. (P) Average stride length and maximum iliac crest height at 21 dpi in mice injected with control or *Prmt6* shRNA AAV (*n* = 5 animals per group). Values are mean ± SEM. **p < 0.01, ***p < 0.001, ****p < 0.0001.

For microglia‐specific *Prmt6* knockdown, we generated pAAV‐Cx3cr1‐EGFP‐miR30shRNA(*Prmt6*) and control pAAV‐Cx3cr1‐EGFP‐miR30shRNA(NC) and injected them into the T10 spinal segment 2 weeks before SCI (Figure 4A). P2Y12 expression was unchanged at the injection sites before injury in both groups (Figure S5), and efficient *Prmt6* knockdown in microglia was confirmed 2 weeks after AAV delivery (Figure 4B,C). At 14 dpi, microglia‐specific *Prmt6* knockdown restored a homeostatic microglial state, with reduced CD68 and increased P2Y12 intensity at the lesion site compared with control AAV (Figure 4D–F). Consistently, microglia‐specific *Prmt6* knockdown led to a smaller lesion area, as demonstrated by HE staining (Figure 4G,H), and to diminished deposition of fibrotic scar components (fibronectin and CSPG) (Figure 4I,J). Perilesional neurons also exhibited more preserved morphology with uniformly distributed Nissl bodies in the microglial *Prmt6*‐knockdown group (Figure 4K). To assess axonal regrowth, propriospinal axons were traced with BDA injected at T7 one week after SCI (Figure 4A). At 21 dpi, BDA‐labelled axons traversed the lesion in mice with microglia‐specific *Prmt6* knockdown but were absent in vector controls (Figure 4L,M). Functionally, *Prmt6* knockdown in microglia improved hindlimb motor function, with greater ankle dorsiflexion, increased stride length, and higher iliac crest position compared with controls (Figure 4N–P).

To further investigate whether *Prmt6* deficiency in blood‐borne cells influences microglial homeostasis after SCI, we transplanted *Prmt6* ^−/−^ bone‐marrow cells (BMCs) into γ‐irradiated wild‐type and *Prmt6* ^−/−^ recipients to establish *Prmt6* ^−/−^→WT and *Prmt6* ^−/−^→*Prmt6* ^−/−^ groups (Figure S6A). Ninety days post‐transplantation, mice underwent T10 crush. Compared with *Prmt6* ^−/−^→*Prmt6* ^−/−^ mice, *Prmt6* ^−/−^→WT mice showed lower P2Y12 intensity and increased numbers of CD68^+^ cells at the lesion (Figure S6B–E), suggesting that only *Prmt6*‐deficiency in blood‐borne cells cannot reestablish local microglial homeostasis. Consistently, *Prmt6* ^−/−^→WT mice displayed a larger fibrotic scar, reduced NF‐H^+^ axonal regrowth, and poorer motor recovery than *Prmt6* ^−/−^→*Prmt6* ^−/−^ controls (Figure S6F–M). Taken together, these findings suggested that *Prmt6* deficiency in microglia, not in blood‐borne cells, was necessary for the restoration of microglial homeostasis.

### 2.4 PRMT6 Inhibitor Administration was Effective Only in Microglia Homeostasis Re‐Establishment Phase

**FIGURE 5:**
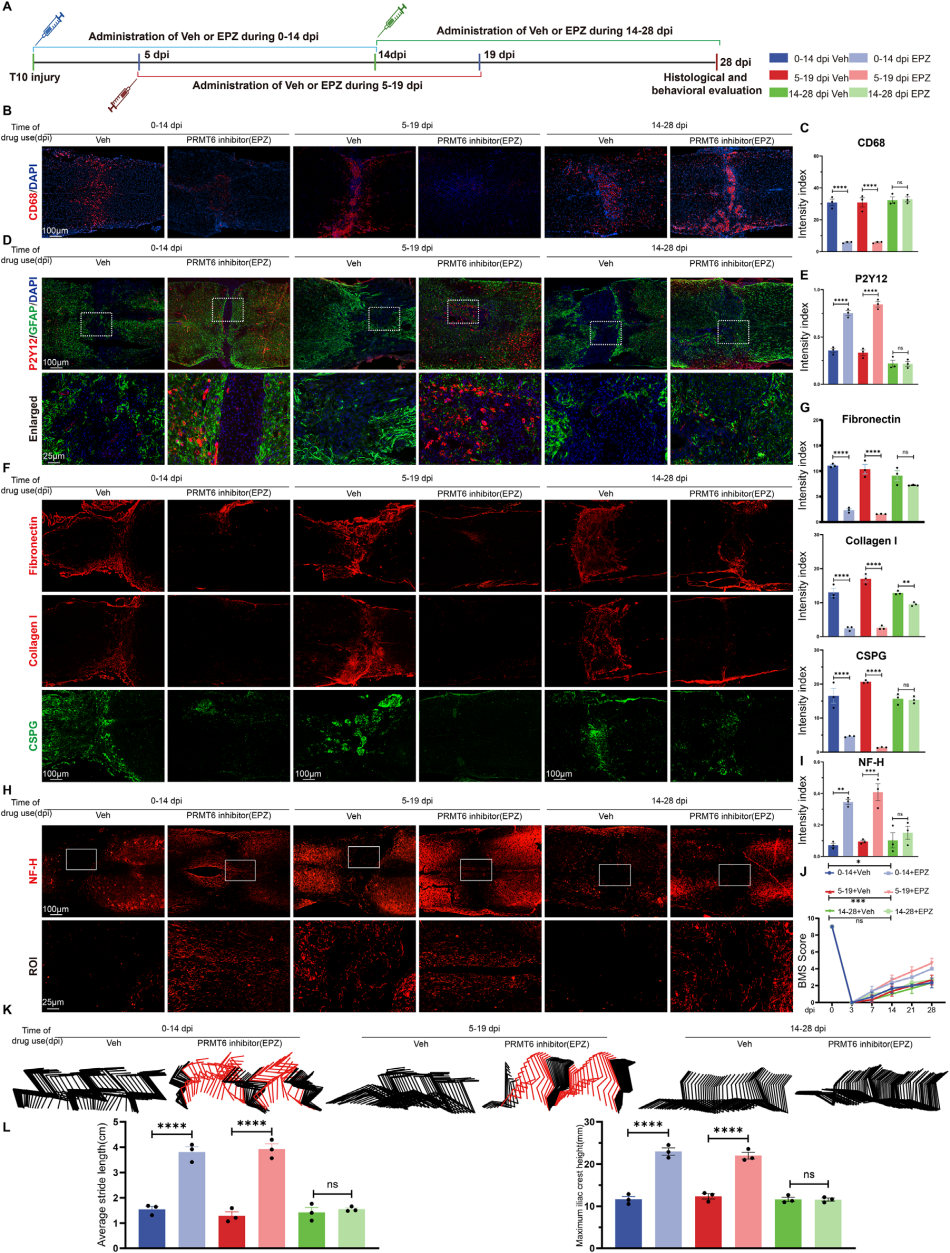
Early PRMT6 inhibition promoted microglial homeostasis and scar‐limited repair after SCI. (A) Timeline of EPZ020411 (PRMT6 inhibitor) or vehicle administration during 0–14, 5–19, or 14–28 dpi. Created in BioRender. Weilin, Peng. (2026) https://BioRender.com/tl2mwmh. (B,C) CD68 immunofluorescence at 28 dpi (B) and quantification of CD68 intensity at lesion sites (C) in the indicated treatment groups (*n* = 3 animals per group). (D,E) P2Y12 and GFAP co‐staining (D) and quantification of P2Y12 intensity at lesion sites (E) at 28 dpi in the indicated treatment groups (*n* = 3 animals per group). Boxed regions are shown at higher magnification. (F,G) Representative images of fibronectin, collagen I, and CSPG staining at 28 dpi (F) and quantification of their intensities at lesion sites (G) in each treatment group (*n* = 3 animals per group). (H,I) NF‐H immunofluorescence within lesion sites at 28 dpi (H) and quantification of NF‐H intensity (I) (*n* = 3 animals per group). (J) BMS scores over 28 days for each group (*n* = 3 animals per group); statistical analysis is performed on scores at day 28. (K) Representative stick diagrams of hindlimb movements at 28 dpi in the different treatment groups. (L) Average stride length and maximum iliac crest height at 28 dpi in the different groups (*n* = 3 animals per group). Values are mean ± SEM. *p < 0.05, **p < 0.01, ***p < 0.001, ****p < 0.0001 (one‐way ANOVA).

To define the therapeutic potential and window of PRMT6 inhibition, we treated mice with EPZ020411, a selective PRMT6 inhibitor, during three post‐injury intervals (0–14, 5–19, and 14–28 dpi), with DMSO given in parallel as vehicle control (Figure 5A).

At 28 dpi, EPZ020411 administration during 0–14 dpi or 5–19 dpi markedly restored microglial homeostasis, as evidenced by reduced CD68 and increased P2Y12 expression at the lesion site (Figure 5B–E), accompanied by decreased deposition of fibrotic ECM components, including fibronectin, collagen I, and CSPG (Figure 5F,G), greater NF‐H^+^ axonal growth across the lesion (Figure 5H,I), and improved locomotor performance in behavioral assays (Figure 5J–L).

In contrast, administration of EPZ020411 at 14–28 dpi did not alter CD68/P2Y12 profiles, fibrotic ECM deposition, NF‐H expression, or functional recovery compared with vehicle‐treated controls (Figure 5B–L).

Together, these data demonstrated that pharmacological PRMT6 inhibition exerted robust pro‐repair effects only when initiated in the early post‐injury phase, indicating a critical therapeutic window during which targeting PRMT6 could re‐establish microglial homeostasis and promote tissue repair after SCI.

### 2.5 *Prmt6* Deficiency Promoted FAO by Upregulating PGC‐1α Transcription in Microglia

**FIGURE 6:**
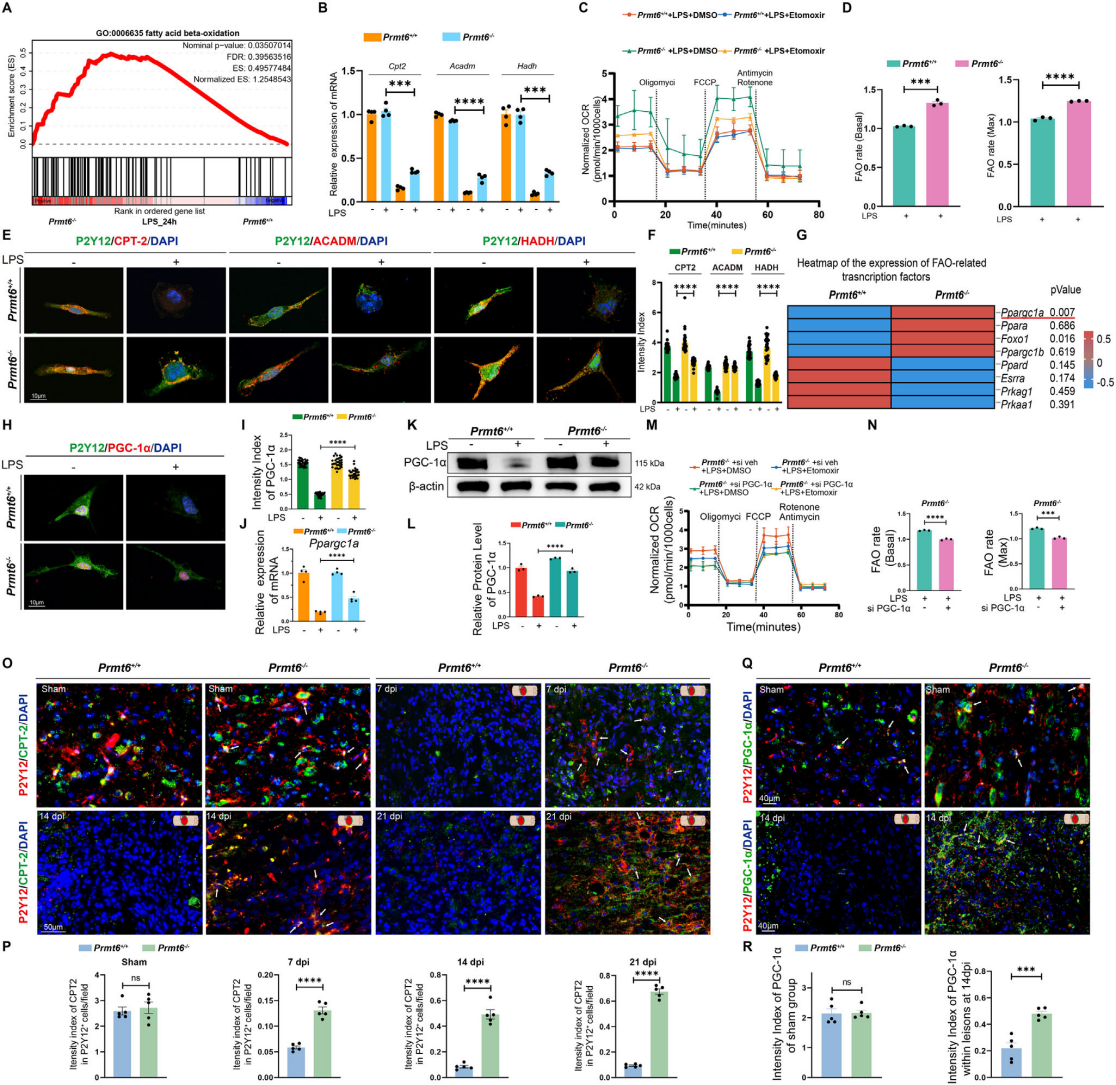
*Prmt6* deficiency enhanced PGC‐1α–dependent FAO in microglia. (A) Gene Set Enrichment Analysis (GSEA) showing enrichment of the FAO pathway in *Prmt6* ^−/−^ versus *Prmt6* ^+/+^ primary microglia after LPS stimulation. (B) qPCR analysis of FAO‐related genes (*Cpt2*, *Acadm*, *Hadh*) in *Prmt6* ^+/+^ and *Prmt6* ^−/−^ microglia after LPS stimulation (*n* = 4 per group). (C) Oxygen consumption rate (OCR) traces before and after etomoxir treatment under basal and FCCP‐stimulated maximum conditions in *Prmt6* ^+/+^ and *Prmt6* ^−/−^ microglia (*n* = 3 per group). (D) FAO rate in *Prmt6* ^+/+^ and *Prmt6* ^−/−^ microglia at basal and maximal respiration, calculated as the ratio of OCR with control treatment to OCR with etomoxir under basal and maximum conditions (*n* = 3 per group). (E,F) Representative immunofluorescence images (E) and quantification (F) of CPT2, ACADM, and HADH in *Prmt6* ^+/+^ and *Prmt6* ^−/−^ primary microglia before and after LPS stimulation (*n* = 30 cells per group). (G) Heatmap of FAO‐related transcription factors in *Prmt6* ^+/+^ and *Prmt6* ^−/−^ microglia after LPS, highlighting *Ppargc1a* as the most significantly upregulated gene. (H,I) Representative images of PGC‐1α co‐stained with P2Y12 in *Prmt6* ^+/+^ and *Prmt6* ^−/−^ primary microglia after LPS stimulation (H) and quantification of PGC‐1α fluorescence intensity (I) (*n* = 30 cells per group). (J) qPCR analysis of *Ppargc1a* mRNA levels in *Prmt6* ^+/+^ and *Prmt6* ^−/−^ primary microglia after LPS stimulation (*n* = 4 per group). (K,L) Western blot (K) and quantification (L) of PGC‐1α protein levels in *Prmt6* ^+/+^ and *Prmt6* ^−/−^ primary microglia after LPS stimulation (*n* = 3 per group). (M,N) OCR traces before and after etomoxir treatment (M) and FAO rate under basal and maximal conditions (N) in *Prmt6* ^−/−^ microglia transfected with siPGC‐1α or control siRNA (*n* = 3 per group). (O) Co‐staining of P2Y12 and CPT2 in intact spinal cord and in lesions at 7, 14, and 21 dpi from *Prmt6* ^+/+^ and *Prmt6* ^−/−^ mice. (P) Quantification of CPT2 intensity in P2Y12^+^ cells in O (*n* = 5 per group). (Q) Representative images of P2Y12 and PGC‐1α co‐staining in spinal cord from *Prmt6* ^+/+^ and *Prmt6* ^−/−^ mice before and 14 days after SCI. (R) Quantitative analysis of PGC‐1α intensity in Q (*n* = 5 per group). Values are mean ± SEM. ***p < 0.001, ****p < 0.0001.

To explore how PRMT6 regulates microglial homeostasis, we established an in vitro activation model using primary microglia for RNA‐seq analysis. LPS‐activated primary microglia recapitulated our in vivo findings: compared with their *Prmt6* ^+/+^ counterparts, *Prmt6* ^−/−^ microglia displayed higher P2Y12 and lower CD68 expression after LPS stimulation (Figure S7). We therefore used this model to investigate the mechanisms by which PRMT6 disrupts microglial homeostasis. Given that metabolic state critically shapes microglial activation and homeostasis [27, 39], we next compared metabolic pathways in *Prmt6* ^−/−^ versus *Prmt6* ^+/+^ microglia after LPS stimulation. KEGG analysis pointed to lipid metabolism as the most prominently altered pathway (Figure S8A), and GSEA revealed a significant enrichment of the FAO program in *Prmt6* ^−/−^ cells (Figure 6A). Consistent with this, FAO‐related genes, including *Cpt2*, *Acadm*, and *Hadh*, were upregulated in *Prmt6* ^−/−^ microglia (Figure S8B), and their corresponding proteins CPT2 (carnitine palmitoyltransferase 2), ACADM (acyl‐coA dehydrogenase medium chain), and HADH (hydroxyacyl‐CoA dehydrogenase) were increased, as shown by qPCR (Figure 6B), western blotting (Figure S8C,D), and immunofluorescence (Figure 6E,F). To more precisely assess the impact of PRMT6 on microglial FAO, we pre‐treated *Prmt6* ^+/+^ and *Prmt6* ^−/−^ microglia with etomoxir, a specific inhibitor of mitochondrial FAO [40]. By comparing OCR values before and after etomoxir, we found that the etomoxir‐induced decrease in OCR (reflecting FAO‐dependent respiration) was significantly greater in *Prmt6* ^−/−^ microglia than in *Prmt6* ^+/+^ cells following LPS stimulation (Figure 6C,D), indicating that loss of PRMT6 enhanced FAO activity and increased the reliance of microglial respiration on FAO. Consistently, blocking FAO with etomoxir in LPS‐treated *Prmt6* ^−/−^ microglia increased CD68 and reduced P2Y12 (Figure S9A–D), suggesting the link between microglial homeostasis restored by *Prmt6* deficiency and FAO. Building on the finding that *Prmt6* deficiency enhances FAO in microglia, we next investigated how PRMT6 controls FAO. RNA‐seq analysis of LPS‐treated microglia revealed altered expression of several FAO‐related transcriptional regulators, including *Ppargc1a*, *Ppara*, *Foxo1*, *Ppargc1b*, *Ppard*, *Esrα*, *Prkag1*, and *Prkaa1* [41, 42]. Among these, *Ppargc1a* was most prominently upregulated in *Prmt6*‐deficient cells and ranked highest by *p* value (Figure 6G). *Ppargc1a* encodes PGC‐1α, a key transcriptional activator of mitochondrial FAO, and we therefore examined its regulation by PRMT6 [43]. In line with the transcriptomic data, *Ppargc1a* mRNA and PGC‐1α protein were increased in *Prmt6* ^−/−^ versus *Prmt6* ^+/+^ microglia after LPS, as shown by qPCR (Figure 6J), immunofluorescence (Figure 6H,I), and western blotting (Figure 6K,L).

To test whether enhanced FAO in *Prmt6* ^−/−^ microglia is PGC‐1α dependent, we performed rescue experiments using siPGC‐1α and control siRNA. Knockdown of PGC‐1α in LPS‐treated *Prmt6* ^−/−^ primary microglia was confirmed by immunofluorescence, qPCR, and western blotting (Figure S10A–E) and led to reduced FAO‐dependent OCR, as assessed by etomoxir‐sensitive respiration (Figure 6M,N), as well as downregulation of FAO enzymes CPT2, ACADM, and HADH (Figure S10D–I). These data indicated that *Prmt6* deficiency promoted microglial FAO in a PGC‐1α‐dependent manner.

We next examined in vivo whether this PGC‐1α‐dependent FAO pathway is restored in homeostatic microglia in *Prmt6* ^−/−^ mice after SCI. In uninjured adult spinal cord, CPT2 immunofluorescence was similar in *Prmt6* ^+/+^ and *Prmt6* ^−/−^ mice (Figure 6O,P). Following SCI, however, CPT2 intensity was markedly higher at lesion sites of *Prmt6* ^−/−^ mice at 7, 14, and 21 dpi, with P2Y12^+^ homeostatic microglia showing pronounced CPT2 upregulation (Figure 6O,P). Likewise, PGC‐1α fluorescence did not differ between *Prmt6* ^+/+^ and *Prmt6* ^−/−^ mice in the intact spinal cord, but at 14 dpi was substantially elevated at the lesion in *Prmt6* ^−/−^ mice relative to their *Prmt6* ^+/+^ counterparts (Figure 6Q,R). Together, these results established that *Prmt6* deficiency engaged a PGC‐1α‐dependent FAO pathway in microglia both in vitro and in vivo.

### 2.6 *Prmt6* Deficiency Restored Microglial Homeostasis via PGC‐1α‐Dependent FAO after SCI

**FIGURE 7:**
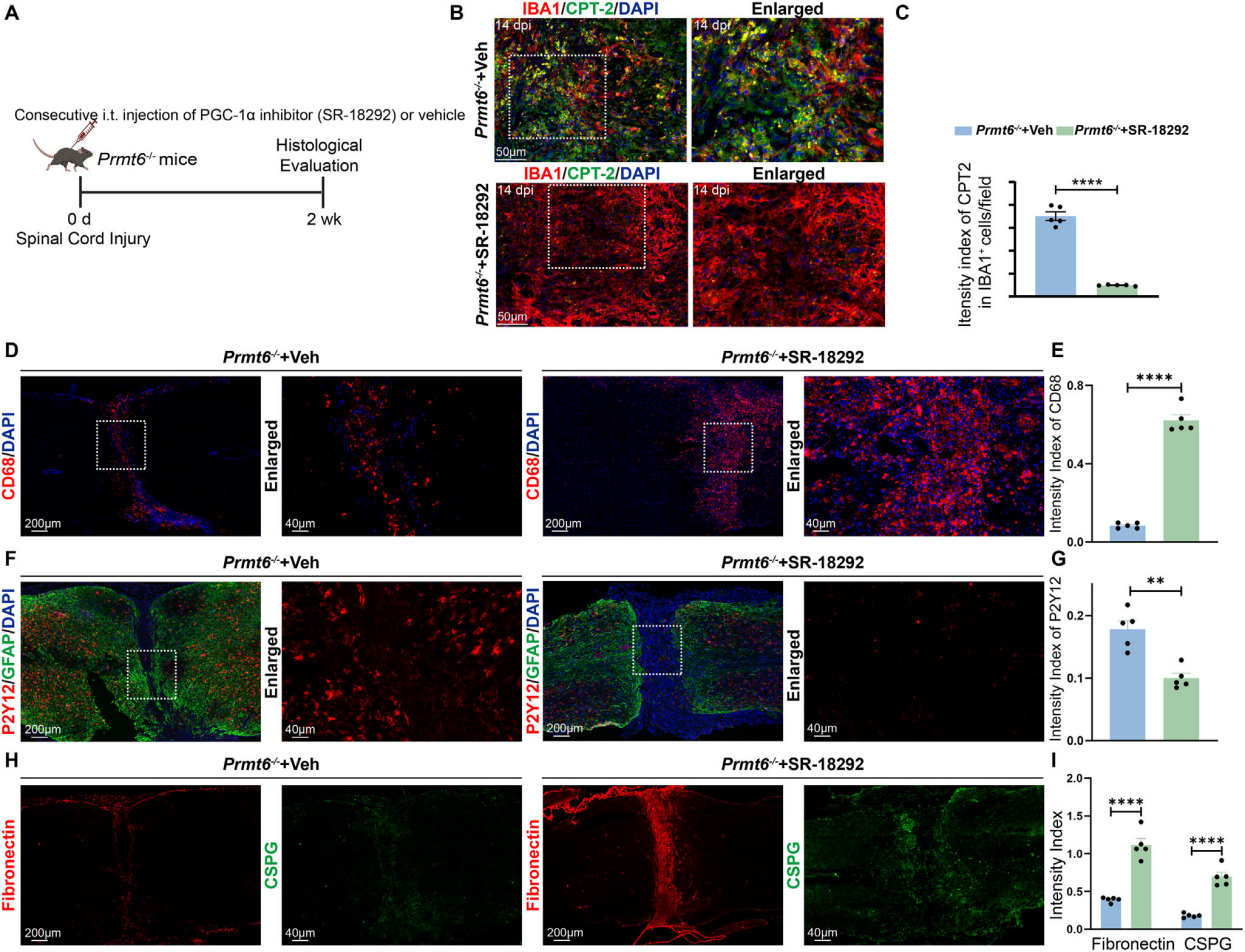
*Prmt6* deficiency restored microglial homeostasis through PGC‐1α‐dependent FAO. (A) Schematic illustration of the experimental design of PGC‐1α inhibitor and vehicle administration. Created in BioRender. Weilin, Peng. (2026) https://BioRender.com/tl2mwmh. (B,C) Representative images of CPT2 and IBA1 co‐staining at 14 dpi (B) and quantification of CPT2 intensity in IBA1^+^ microglia at lesion sites (C) in *Prmt6* ^−/−^ mice treated with SR‐18292 or vehicle (*n* = 5 animals per group). Boxed regions are shown at higher magnification. (D,E) Representative images of CD68 staining at 14 dpi (D) and quantification of CD68 intensity at lesion sites (E) in *Prmt6* ^−/−^ mice treated with SR‐18292 or vehicle (*n* = 5 animals per group). Boxed regions are shown at higher magnification. (F,G) Representative images of P2Y12 staining at 14 dpi (F) and quantification of P2Y12 intensity at lesion sites (G) in *Prmt6* ^−/−^ mice treated with SR‐18292 or vehicle (*n* = 5 animals per group). Boxed regions are shown at higher magnification. H, I) Representative images of fibronectin and CSPG staining at 14 dpi (H) and quantification of fibronectin and CSPG intensity at lesion sites (I) in *Prmt6* ^−/−^ mice treated with SR‐18292 or vehicle (*n* = 5 animals per group). Values are mean ± SEM. **p < 0.01, ***p < 0.001, ****p < 0.0001.

Building on the identification of a restored PGC‐1α‐dependent FAO in *Prmt6*‐deficient microglia, we next tested whether this pathway is required for the restoration of microglial homeostasis. Adult *Prmt6* ^−/−^ mice received daily intrathecal injections (i.t.) of SR‐18292, a PGC‐1α inhibitor, after SCI (Figure 7A). Compared with vehicle‐treated *Prmt6* ^−/−^ mice, SR‐18292 administration markedly reduced CPT2 immunofluorescence in microglia at the lesion site, indicating effective disruption of PGC‐1α‐driven FAO (Figure 7B,C). At 14 dpi, PGC‐1α inhibition increased CD68 and decreased P2Y12 expression in *Prmt6* ^−/−^ lesions, signifying a failure to maintain the restored microglial homeostatic phenotype, and these changes were accompanied by broader and more intense deposition of fibrotic ECM components such as fibronectin and CSPG, which indicated impaired scar‐limited repair (Figure 7D–I). *i*, siPGC‐1α in LPS‐treated *Prmt6* ^−/−^ microglia abolished the *Prmt6*‐deficiency‐induced homeostatic profile, increasing CD68 and reducing P2Y12 (Figure S10J–M).

Together with the preceding FAO data, these findings demonstrated that the ability of *Prmt6* deficiency to restore microglial homeostasis relied on PGC‐1α‐dependent FAO.

### 2.7 PRMT6 Suppressed PGC‐1α Expression by Asymmetrically Dimethylating the H3R2 at *Ppargc1a* Promoter

**FIGURE 8:**
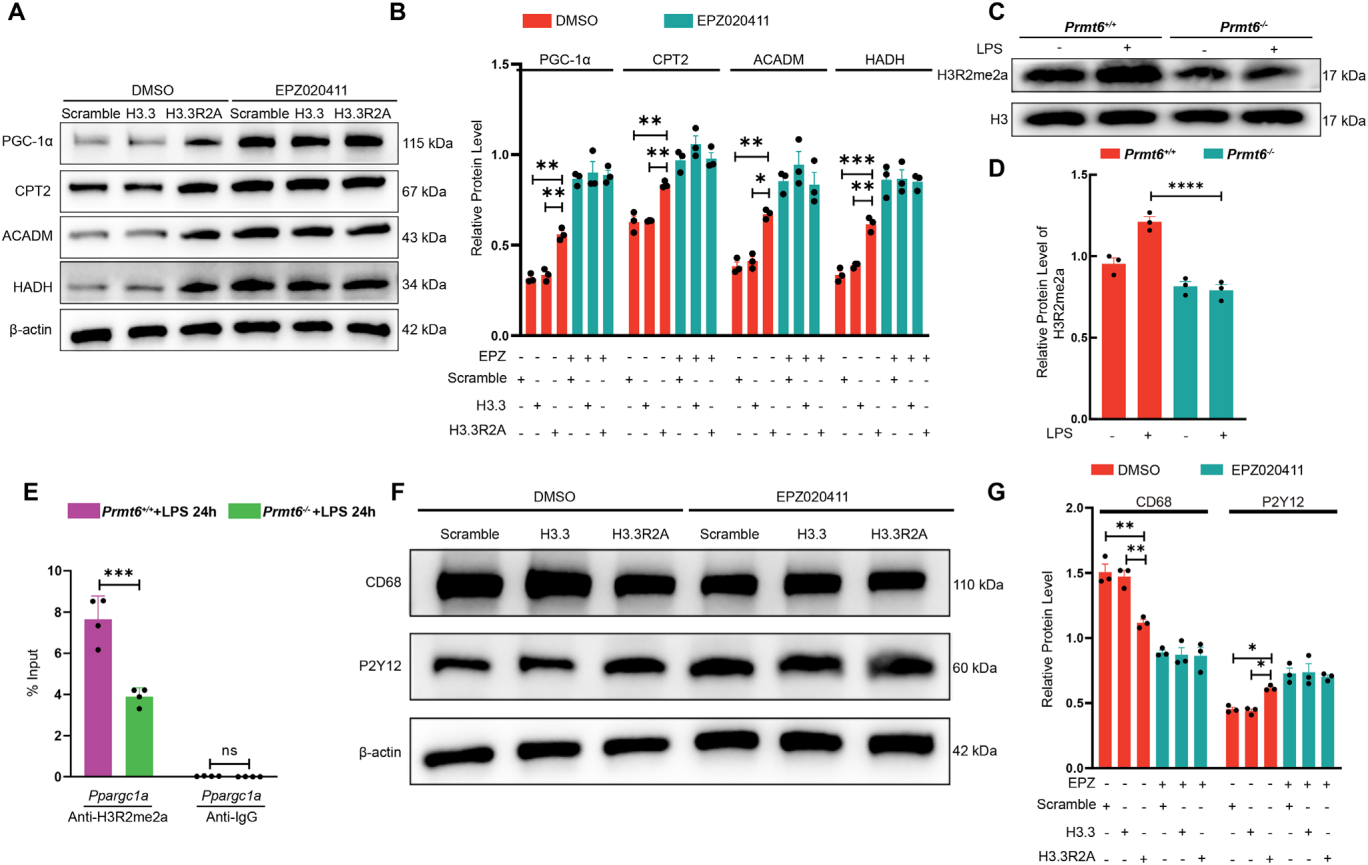
PRMT6 suppressed FAO in activated microglia via methylation of H3R2 at the *Ppargc1a* promoter. (A,B) Western blot (A) and quantitative analysis (B) show that overexpression of H3.3R2A, which inhibits methylation at the H3R2 residue, significantly increased the levels of PGC‐1α and its downstream FAO enzymes in LPS‐treated BV‐2 cells (*n* = 3 per group). (C,D) Western blot (C) and quantitative analysis (D) of H3R2me2a indicate that *Prmt6* deficiency counteracted LPS‐induced upregulation of H3R2me2a modification in primary microglia (*n* = 3 per group). (E) ChIP‐qPCR analysis reveals that *Prmt6* deficiency decreased the repressive H3R2me2a modification at the promoter of *Ppargc1a* (*n* = 4 per group). (F,G) Western blot (F) and quantitative analysis (G) show that overexpression of H3.3R2A, which blocks methylation at the H3R2 site, significantly restored microglial homeostatic marker P2Y12 and decreased the activation marker CD68 in LPS‐treated BV‐2 cells (*n* = 3 per group). Values are mean ± SEM. *p < 0.05, **p < 0.01, ***p < 0.001, ****p < 0.0001.

Given that H3R2me2a, a target for PRMT6 catalysis, serves as a repressive histone modification that decreases chromatin accessibility of target genes [26, 44], we constructed a histone plasmid with an arginine‐to‐alanine mutation at the H3.3R2 residue (H3.3R2A) to competitively inhibit methylation at the H3R2 site [26]. In LPS‐treated BV‐2 cells, inhibition of H3R2 methylation via overexpression of H3.3R2A upregulated protein levels of PGC‐1α and its downstream targets (CPT2, ACADM, and HADH). This effect was nullified by PRMT6 inhibition, indicating that PRMT6 suppresses PGC‐1α‐mediated FAO through H3R2me2a modification (Figure 8A,B). Moreover, in *Prmt6* ^−/−^ primary microglia, the protein level of H3R2me2a was significantly lower than that in *Prmt6* ^+/+^ microglia after LPS stimulation (Figure 8C,D). ChIP‐qPCR further validated a significant reduction in H3R2me2a modification at the *Ppargc1a* promoter in *Prmt6* ^−/−^ cells, suggesting increased chromatin accessibility for *Ppargc1a* (Figure 8E). Consequently, H3.3R2A overexpression significantly reduced the protein level of CD68 and elevated that of P2Y12 (Figure 8F,G). Collectively, these results confirmed that PRMT6 suppressed the transcription of PGC‐1α by methylating H3R2 at its promoter following LPS stimulation, enhancing our understanding of its epigenetic role in the regulation of PGC‐1α‐mediated FAO and microglial activation.

## 3 Discussion

Unlike the scar‐free repair observed in neonatal mice, where microglial homeostasis is restored after SCI, adult microglia at lesion sites fail to re‐establish a functionally homeostatic state characterized by high P2Y12 and low CD68 expression, leading to chronic neuroinflammation, impaired axonal regeneration, and scar formation [6]. However, the mechanisms underlying this failure remain unclear. Our study provides novel insights into the role of PRMT6 in disrupting the re‐establishment of microglial homeostasis following adult SCI. To hone in on key genes regulating chronic microglial activation post‐SCI, we focused on GO terms related to bioprocess associated with microglia. Initially, given that spinal cord injury induces acute and chronic stress modulating microglial response [45, 46], the term “Cellular response to stress” was selected. Second, considering that microglia activation includes obvious changes in the organization of organelles and cellular metabolism [27, 28, 47], the GO term “Regulation of organelle organization” and “Regulation of cellular metabolic process” were elected. Furthermore, given that recent study underlined the crucial role of transcriptional pause/release mediated by RNA polymerase (Pol) II in controlling the function of myeloid cells during tissue injury and inflammation [29], the GO term “Regulation of transcription by RNA polymerase II” was selected. To further identify the regulatory upstream genes, another three terms: “Regulation of signal transduction”, “Regulation of gene expression”, and “Biological regulation” were added for Venn diagram. Through RNA‐seq analysis and subsequent immunofluorescence, we identified that PRMT6 was upregulated in CD68^+^ and IBA1^+^ cells both at the lesion border and core where microglia and blood‐borne macrophages reside at, respectively [48], at the lesion sites of adult mice at 3, 7, and 14dpi, as well as in activated primary microglia. However, this upregulation was not observed in GFAP^+^ astrocytes or NeuN^+^ neurons at 3, 7, 14, and 21 dpi at the lesion site, suggesting that the increased expression of PRMT6 after adult SCI might mainly attributed to activated myeloid cells. Notably, PRMT6 expression remained a relatively low level in neonatal microglia before and after SCI, suggesting its involvement in the failure to restore microglial homeostasis in adult SCI.

Disruption of microglial homeostasis contributes to the progression of various CNS disorders. *Tgfb1‐*deficiency in microglia disrupts their homeostatic state and leads to their activation, which causes cognitive deficits in adult mice [49]. In Alzheimer's disease, the reduced expression of homeostatic microglial genes is correlated with neuronal loss [50]. In spinal cord injury, Li et al. [12] show that in adult mice, regeneration‐promoting microglia identified in neonatal SCI, although still present in the spinal cord before SCI, were less abundant, emerged later, and expressed higher levels of *Cd68* and lower levels of *P2ry12* compared to neonates after SCI, indicative of reduced regeneration‐promoting power. After SCI, *Prmt6* ^−/−^ mice displayed earlier re‐emergence of P2Y12^+^ and TMEM119^+^ microglia by 3 dpi at the lesion edge, with sustained higher counts at 7, 14, and 21 dpi. These findings suggested that *Prmt6* deficiency accelerated the re‐establishment of a homeostatic state in microglia, indicated by restored P2Y12 and suppressed CD68 profiles, thereby promoting their population at the injury site after adult SCI.

Accumulating evidence identifies activated microglia as essential organizers of the astroglia‐derived scar [51, 52]. Bellver‐Landete et al. showed that pharmacological depletion of microglia produces a disorganized glial scar in an IGF‐1‐dependent manner, supporting the view that astrocytes require microglia‐derived cues to assemble a structured border around the lesion [48]. Liddelow et al. further demonstrated that microglia‐derived cytokines, including IL‐1α, TNF, and C1q, are sufficient to drive astrocytes toward a reactive and neurotoxic state [53], providing a mechanistic framework for microglia‐to‐astrocyte instruction after CNS injury. In intracerebral hemorrhage models, microglial activation has also been linked to astrocyte proliferation and subsequent scar formation across both acute and chronic phases, indicating that this organizing role of microglia extends beyond SCI [54].

In neonatal mice, Li et al. reported a scar‐free repair phenotype that correlates with the rapid re‐emergence of homeostatic microglial programs after injury [6]. Within this context, microglial depletion via PLX3397 or *Csf1r* knockout leads to persistent astrocyte‐derived scarring and failure of scar‐free resolution, placing microglia as a necessary component of the regenerative repair trajectory in neonates. These observations collectively support a model in which the presence of microglia, together with their injury‐stage‐dependent functional state, shapes astrocytic behaviors that underlie scar formation.

In adult SCI, astrocytes form the principal cellular substrate of the scar, while microglia contribute to the injury milieu that instructs astrocytic proliferation, border assembly, and extracellular matrix deposition [54, 55]. In this multi‐factorial setting, PRMT6 functions as one of the key gatekeepers of microglial state programs and thereby influences scar formation by remodeling the inflammatory cue landscape perceived by astrocytes. Consistent with this framework, the reduction of astroglia‐derived scarring observed after microglial *Prmt6* depletion aligns with the concept that restoration of microglial homeostatic programs constrains the chronic inflammatory signals that sustain dense scar formation around the lesion. At the same time, the molecular determinants that link discrete microglial functional states to specific astrocytic scarring programs remain to be delineated. The microglia‐astrocyte coupling provides a tractable direction for future therapeutic strategies aimed at promoting regeneration after SCI.

Fibrotic scar formation in the central nervous system is closely associated with the local inflammatory milieu [56]. After adult SCI, chronically activated microglia and macrophages play crucial roles in shaping the inflammatory microenvironment. In adult mice transplanted with neonatal microglia or treated with protease inhibitors that resolve local neuroinflammation, a significant reduction in CD68 intensity and ECMs deposition was observed, along with restored microglial homeostasis [6, 12]. In this study, we showed that *Prmt6* ^−/−^ mice exhibited a significant reduction in the intensity of CD68 at the lesion site at 7,14, and 21dpi, indicating alleviated neuroinflammation. Moreover, these mice showed a reduction in scar tissue size and ECMs components (CSPG, fibronectin, and collagen I), potentially creating a permissive environment for axonal regeneration.

Consistent with the restoration of microglial homeostasis and scar‐limited repair, *Prmt6* deficiency fostered a profoundly neuroprotective and pro‐regenerative environment. This was evidenced by the preserved morphology of perilesional neurons, robust axonal regeneration, and an increased density of MAP2‐positive neuronal structures, which collectively provided the structural foundation for functional recovery. We acknowledge that the MAP2 signal alone cannot distinguish between the dendritic remodeling of surviving neurons and neurogenesis. Future lineage tracing studies are therefore required to dissect these respective contributions and to elucidate the precise mechanisms by which P2Y12^+^ homeostatic microglia orchestrate this neuronal repair.

During our review, recent findings have emphasized the role of microglial homeostasis in repairing spinal cord injury. Li et al. found that inhibiting microglia by Adrb2 agonist could remodel the scar microenvironment, permitting the regeneration of axons [57]. Overexpressing FBXL12 promoted and maintained a “scar‐less healing” phenotype, reducing local ECMs deposition [32]. Taken together, our study and these recent findings demonstrate the fact that restoring the population of P2Y12^+^ microglia is critical in modulating the microenvironment after spinal cord injury and chronic scar‐forming. However, this study investigated the topic of microglial homeostasis from the immunometabolism perspective, contributing to the epigenetic regulation of microglial fatty acids oxidation.

Distinguishing the role of microglia and macrophages in SCI is a crucial issue, which could enhance our understanding of their similarities and differences in neuroinflammation [58, 59]. By inoculating AAV with *Cx3cr1* promoter into the spinal cord before SCI, *Prmt6* was specifically knocked down in local microglia, leading to significant restoration of microglial homeostasis and scar‐limited repair. Our bone marrow transplantation experiments determined that *Prmt6* deficiency in blood‐borne macrophages could alleviate local inflammation, reduce chronic scarring and promote axonal regrowth, which suggested that PRMT6 might be a critical mediator in inflammatory responses induced by myeloid cells. Further studies are needed to investigate the effect of *Prmt6* deficiency in other inflammatory disease models.

Considering that PRMT6 was remarkably upregulated in microglia before 14 dpi, we examined the effective treatment window of PRMT6 inhibitor. PRMT6 inhibition during 0–14 and 5–19 dpi effectively promoted microglial homeostasis and reduced the area of ECMs, enhancing axonal regrowth and promoting motor function. Previous behavioral studies demonstrated that motor function continuously increased until day 14, and after 14 dpi, the speed of functional recovery kept a low rate [12, 60]. This also explains why administration of PRMT6 inhibition before 14 dpi could serve the most effective therapeutic power. Similarly, compared with delayed administration, Li et al. demonstrated that the injection of Adrb2 agonist at acute stage after SCI showed better effectiveness in promoting functional recovery [57]. Collectively, our study and Li's work lay a solid foundation for the early intervention of microglial homeostasis restoration in repairing spinal cord injury.

To rule out the possibility that the observed effects of *Prmt6* deficiency were compensated by other protein arginine methyltransferases, we assessed the expression of key PRMT family members (*Prmt1*, *Prmt2*, *Prmt3*, *Prmt4*, *Prmt5*, and *Prmt8*) in activated primary microglia. Our data confirmed that their expression remained unaltered despite *Prmt6* deficiency (Figure S10), thereby strengthening the conclusion that the phenotypic outcomes are specifically attributable to the loss of PRMT6.

Recent studies support the fact that FAO is also present in microglia. The Mito Fuel Flex Test demonstrated microglial reliance on fatty acids under basal conditions [61]. In experiments where the optic nerve was incubated in artificial cerebrospinal fluid (aCSF), the populations of microglia remained stable, while more than 70% of astrocytes did not survive after 24 h in glucose‐free medium [62]. These results suggest that FAO may play a critical role in modulating microglial metabolic landscape and homeostasis. PGC‐1α is a crucial regulator of fatty acid oxidation, mitochondrial biogenesis, and respiration [43]. In LPS‐induced neuroinflammation, resveratrol reprograms microglia from a pro‐inflammatory to an anti‐inflammatory state by elevating PGC‐1α levels [63]. In acute ischemic stroke (AIS), PGC‐1α reduces neuroinflammation by enhancing microglial mitophagy [64]. However, the effect of PGC‐1α‐mediated FAO in microglia after SCI remains unclear. In the present study, we shown that *Prmt6* deficiency restored PGC‐1α–regulated FAO in microglia following LPS stimulation, an effect observed in vitro and recapitulated in *Prmt6* ^−/−^ mice in vivo. Functionally, pharmacological inhibition of FAO with etomoxir or knockdown of PGC‐1α abolished the *Prmt6* depletion‐induced re‐expression of the homeostatic microglial marker P2Y12. Consistently, in *Prmt6* ^−/−^ mice, PGC‐1α inhibition reduced the FAO enzyme CPT2, prevented P2Y12 re‐emergence, and elevated the activation marker CD68, accompanied by a shift toward scar‐based repair. Together, these rescue experiments position the PGC‐1α‐FAO module as a key downstream effector through which PRMT6 shapes microglial state programs, supporting microglial immunometabolism as a tractable entry point for promoting homeostatic features after SCI. Recent evidence supports a promising role for this axis in microglial state control [65, 66]. In a neuropathic pain model, microglial ChREBP activated PGC‐1α transcription to enhance FAO and promote an anti‐inflammatory phenotype; PGC‐1α overexpression rescued the effects of ChREBP knockdown, and etomoxir attenuated this protection, supporting PGC‐1α‐dependent FAO as a driver of microglial programs [65]. In conjunction with our rescue data in SCI models, these observations underscore the PGC‐1α‐FAO axis as a regulatory hub linking microglial metabolism to functional state control. At the level of regulatory hierarchy, PRMT6 operates upstream of this metabolic effector module. By depositing the repressive H3R2me2a modification at the *Ppargc1a* promoter, PRMT6 constrains PGC‐1α availability and thereby limits the FAO program that supports homeostatic microglial features. This positions PRMT6 as an epigenetic gatekeeper coupling chromatin state to immunometabolic outputs during SCI. At the same time, the therapeutic efficacy and optimal treatment window of directly modulating PGC‐1α‐dependent FAO after SCI, as well as the broader regulatory inputs converging on this module, remain to be defined. Future studies examining how these mechanisms interface with PGC‐1α‐dependent FAO across distinct stages of SCI will further clarify how microglial state trajectories can be harnessed to support repair.

Previous studies have established that pro‐inflammatory activation of myeloid cells is typically accompanied by a metabolic switch from oxidative phosphorylation and FAO toward enhanced glycolysis [67], whereas promoting FAO can drive these cells toward a less inflammatory phenotype. In this context, together with a previous report linking PRMT6 to pro‐inflammatory microglial activation through HIF‐1α‐associated metabolic pathways, our present study demonstrated that *Prmt6* deficiency restored PGC‐1α‐driven FAO that promoted microglial homeostasis in adult SCI, supporting the notion that PRMT6 functions as one of the upstream regulators of microglial immunometabolism. Importantly, our findings position PRMT6 as one of the key regulatory gatekeepers that, alongside other microenvironmental cues, modulates the phenotypic switching between microglial homeostatic and activated states. Accordingly, targeting PRMT6 may represent an effective strategy to rebalance microglial metabolism and neuroinflammation.

Epigenetic modifications on histones are key regulators of microglial homeostasis [16, 68, 69]. Recent study reports that mutations in histone H3.3G34R disrupt H3K36me2a, impair DNA methyltransferase DNMT3A recruitment and lead to the accumulation of dyshomeostatic microglia, contributing to neurodegeneration [70]. Furthermore, microglia possess various epigenomic and associated transcriptomic signatures throughout life, including microglial development and aging [17, 18], indicating that the epigenetic heterogeneity between neonatal and adult microglia may influence their ability to restore homeostasis after SCI. Initially, given that PRMT6 could asymmetrically methylate H3R2 [26, 44], we examined the level of H3R2me2a. Consistent with previous studies, *Prmt6* deficiency significantly downregulated this repressive histone modification. Further ChIP assays confirmed the reduced presence of H3R2me2a at the *Ppargc1a* promoter in *Prmt6* ^−/−^ microglia. Additionally, inhibiting H3R2 methylation significantly rescued the decreased PGC‐1α levels and the downregulation of homeostatic marker P2Y12. In contrast, inhibition of PRMT6 simultaneously nullified this effect, suggesting that PRMT6 regulated PGC‐1α in a H3R2me2a‐dependent manner. Our study revealed the epigenetic mechanism by which PRMT6 mediates the transcriptional regulation of PGC‐1α in microglia, enhancing our understanding of the epigenetic regulation conducted by PRMT6 in microglia. To note, though our in vivo data demonstrated the restoration of microglial homeostasis and its metabolic mechanism, it is important to note that the primary microglial culture model used here for exploration, despite employing optimized media conditions, cannot fully capture the complex morphological and functional features of homeostatic microglia within their native tissue environment. While our study delineated the role of the PRMT6/H3R2me2a/PGC‐1α axis in microglial homeostasis, future studies utilizing advanced delivery systems that minimize cellular activation will be essential to definitively validate the function of H3R2me2a in primary microglia and in vivo.

## 4 Conclusion

In conclusion, our study identifies differential PRMT6 expression between neonatal and adult mice before and after SCI, highlighting PRMT6 as one of the key regulatory gatekeepers in microglial homeostasis imbalance in adult SCI. We demonstrate that *Prmt6* deficiency or inhibition effectively reestablishes microglial homeostasis and promotes a scar‐limited repair. Mechanistically, PRMT6 promotes asymmetric dimethylation of the H3R2 residue at the *Ppargc1a* promoter, reducing chromatin accessibility for PGC‐1α and disrupting PGC‐1α‐regulated fatty acid oxidation, which is crucial for maintaining microglial homeostasis.

## 5 Experimental Section

### 5.1 Animals and Ethics Statement

All animal experiments were conducted in accordance with the National Institutes of Health Guide for the Care and Use of Laboratory Animals, and approved by the Ethics Committee of Medicine, Naval Medical University (Approval No. 20240305042940932_0071). Pregnant and nonpregnant female C57BL/6 mice were sourced from the Experimental Animal Center of Naval Medical University (Shanghai, China). Mice purchased were allowed to habituate for at least one week in the animal facility prior to experimentation. The generation and identification of wild‐type (*Prmt6* ^+/+^) and *Prmt6*‐deficiency (*Prmt6* ^−/−^) mice were carried out as previously described (Figure S12) [26]. All mice were bred and housed under specific‐pathogen‐free conditions, including a room temperature of 24°C, 50% humidity and a 12/12‐h light/dark cycle. Mice had ad libitum access to food and water. We made every effort to minimize the number of animals used in the study and to reduce their suffering.

### 5.2 Neonatal Surgery

As demonstrated before [6], at postnatal day 2 (P2), neonatal mice were anesthetized with inhaled isoflurane in oxygen‐enriched air. A laminectomy was performed at the T10 vertebra to expose the spinal cord, which was crushed for 2 s using 0.1 mm‐wide forceps. In the sham group, only the laminectomy was performed without spinal cord injury. Muscles were sutured, and the skin was closed. Mice were warmed for 30–60 min in a box containing original bedding. The surgical site was rubbed with maternal feces using a Q‐tip before returning the pups to the mother. A Nutra‐Gel diet (Bio‐Serv) was provided to prevent cannibalism, and bladder emptying was conducted once a day until sacrifice.

### 5.3 Adult Surgery

8‐weeks‐old female mice weighing 18–20 g were allocated to each group randomly. All surgeries on mice were performed under general anesthesia with 2% inhaled isoflurane in oxygen‐enriched air, using rodent stereotaxic apparatus (David Kopf). AAV injections for *Prmt6* knockdown and control were conducted two weeks before SCI to allow time for molecular expression at the planned SCI location after laminectomy of a single vertebra (Figure 4A). Spinal injections were conducted as previously described [71]. AAVs were injected into two sites (one on each side of the cord, 0.25 µL per side) 0.5 mm below the surface at 0.1 µL per minute, using a pulled calibrated glass pipette and a single‐channel microinjection‐withdrawal pump (KD Scientific). The glass pipette was retracted 10 min after injection. AAVs used in this study include: pAAV‐Cx3cr1 promoter‐EGFP‐3xFLAG‐WPRE (1 × 10^13^ genomic copies per ml in sterile saline) and pAAV‐Cx3cr1 promoter‐EGFP miR30shRNA(NC)‐WPRE (1 × 10^13^ genomic copies per ml in sterile saline), which were designed and generated by Obio Technology (Shanghai, China). For spinal cord injury, a laminectomy was performed at the T10 vertebra to fully expose the spinal cord, followed by a crush for 2 s using 0.1 mm‐wide forceps (Figure S1A). In the sham group, only the laminectomy was conducted without spinal cord injury. After surgery muscles and skins were closed in layers. Mice were placed on a warming pad until fully awake and received buprenorphine twice daily for pain relief for three days. Daily monitoring included checking for infection, abnormal wound healing, and weight loss. Bladder expression was performed two times a day until bladder function was restored. At 1week post‐SCI, tract‐tracing of propriospinal axons was performed by injection of biotinylated dextran amine 10 000 (10% w/v in sterile saline, BDA, Cat# N7167, Sigma, USA) and AAV9 (ssAAV‐hSyn‐mCherry‐WPRE‐SV40pA, 1 × 10^13^ genomic copies per ml in sterile saline, PackGene, Shanghai, China) into the T7 segment after laminectomy of a single vertebra [72]. BDA and ssAAV‐hSyn‐mCherry‐WPRE‐SV40pA was injected into 2 sites (one on each side of the spinal cord, 0.4 µL per side), using a pulled calibrated glass pipette. The glass pipette was retracted 10 min after injection. For the consecutive 14‐days intrathecal (i.t.) injection of SR‐18292 (60 µg/5 µL, Cat# HY‐101491, MedChemoExpress, USA), intrathecal catheterization was performed immediately after SCI [73]. After the process, the catheter was fixed by local muscles. Then, guide the catheter through a subcutaneous tunnel at the neck. For the administration of PRMT6 inhibitor EPZ020411, given that previous study have proven the ability of EPZ020411 (Cat#HY‐12970, MedChemoExpress, USA) to penetrate the blood‐brain barrier (BBB) [21, 23], it was administered via intraperitoneal injection (10 mg/kg) once a day at 0–14, 5–19, and 14–28 dpi. Dimethyl sulfoxide (DMSO, Cat# HY‐Y0320, MedChemoExpress, USA) was used as a control and administered at the same dose.

### 5.4 Behavioral Analysis

The Basso Mouse Scale (BMS) open field test and Louisville Swim Scale were used to assess motor function after injury, as previously described [38]. Mice were observed by two independent investigators in a double‐blinded manner at 3, 7, 14, and 21 dpi. For mice treated with EPZ020411 or vehicle, the observation period was extended to 28 dpi. For gait analysis, mice were placed on the treadmill (100 cm long and 4 cm wide) with markers on iliac crest, hip, knee, ankle, and toe after bladder emptying. The movement was recorded using a Nikon camera. If the hindlimb did not move, the length of the forelimb movement was considered as the stride length. The maximal iliac crest height and stride length were analyzed in a double‐blinded manner, and the stick diagram of hindlimb movements was plotted using MATLAB.

### 5.5 Perfusion and Immunohistochemistry

After inhalation of a lethal dose of isoflurane, mice were perfused transcardially with PBS followed by 4% paraformaldehyde (PFA). The spinal cord was submerged in 4% PFA at 4°C overnight and then incubated in 30% sucrose in PBS for 3 days at 4°C. Once fully dehydrated, a 10 mm segment of the spinal cord centered on the lesion was embedded in optimal cutting temperature compound (OCT) on dry ice. Sagittal sections were cut using a cryostat (Leica) at a thickness of 10 µm and stored at −20°C. Before staining, sections were pre‐warmed to room temperature and dried on a 37°C slide warmer for 2 h. After washing with PBS for three times, sections were treated with a blocking solution containing 10% donkey serum (Abcam, #ab7475) and 0.2% Triton X‐100(Sigma, #93443) for 1 h at room temperature. Sections were then incubated with primary antibodies (Table S1) at 4°C overnight, followed by a 2 h incubation at room temperature with fluorescent‐labeled secondary antibodies (Table S1). Cell nuclei were stained with DAPI for 30 s. For hematoxylin and eosin (HE) and Nissl staining, paraffin‐embedded sections were first deparaffinized in xylene and rehydrated through a graded ethanol series. After being rinsed thoroughly with distilled water, the sections were processed for staining according to the following procedures. For HE staining, the sections were stained with hematoxylin for 5 min, differentiated in 1% acid alcohol, and blued in ammonia water, followed by counterstaining with 0.5% eosin for 1 min. For Nissl staining, the sections were stained with 0.5% cresyl violet solution for 10–15 min, rinsed briefly in distilled water, and then differentiated in 95% ethanol containing a few drops of acetic acid. After dehydration through a graded ethanol series, the sections were cleared in xylene and mounted with a neutral resin.

### 5.6 Confocal and Widefield Microscopy

Images were acquired using a fluorescent slide scanner (Pannoramic 250FLASH, 3DHISTECH) and confocal microscopy (Cytation C10, BioTek) with the Gen5 software. All images were acquired with identical microscope settings (laser power, gain, and exposure).

### 5.7 Lesion Analysis and Quantification

The lesion site in the spinal cord was anatomically defined and traced using multiple markers, including collagen I, fibronectin, GFAP, P2Y12, CD68, CSPG, and NF‐H, as well as AAV‐Syn‐mCherry and BDA labeling to delineate axons. For each animal, coronal sections spanning the lesion epicenter were collected and processed in parallel across groups. The lesion area (mm^2^) indicated by HE staining was measured using ImageJ software. Immunostaining intensity in the lesion site (200‐µm‐width region centered on the lesion epicenter) was measured using ImageJ software and normalized to the intact (proximal) region of the spinal cord within the same section. Within the lesions, ROIs (regions of interest) were selected to include both the lesion core and the lesion border, where microglia are predominantly distributed. In each section, ROIs were selected to provide detailed visualization of microglial morphology and therefore included both the lesion epicenter and the lesion border, where ramified and activated microglia are most prominently distributed. The same anatomical positions relative to the lesion center and border were used for all animals within each experimental group. High‐magnification images (e.g., 20× or 40× confocal) were then acquired from these predefined ROIs using identical acquisition settings across groups.

### 5.8 Production of Chimeric Mice

The production of bone marrow chimeras was conducted as previously described [48]. In brief, as shown in Figure S6A, *Prmt6* ^+/+^ (wild‐type) and *Prmt6* ^−/−^ mice were exposed to whole‐body γ‐irradiation with a single dose of 7.5 Gy using a cesium‐173 source (Naval Medical University, Shanghai, China) to deplete hematopoietic stem cells (HSCs). Recipients were then injected via the tail vein with 1 × 10^7^ bone marrow cells (BMCs) freshly isolated from either *Prmt6* ^+/+^ or *Prmt6* ^−/−^ donors. BMCs were collected from the femurs and tibias of euthanized donors using Hank's Balanced Salt Solution (HBSS, Gibco) without Ca^2^ *^+^*/Mg^2^ *^+^* and 2% fetal bovine serum (FBS, HyClone), followed by a 25‐gauge needle aspiration. Post‐transplantation, mice were kept in germ‐free cages and fed antibiotics for 2 weeks (2.5 mL of Septra in 200 mL of drinking water). After an additional 3 months (total of 90 days), mice underwent SCI surgery.

### 5.9 Primary Microglia and BV‐2 Cell Culture

Primary microglia cultures were prepared as previously described [74]. Briefly, as demonstrated in Figure S1F, neonatal mice(P0‐2) were euthanized with a lethal dose of inhaled isoflurane, and brains were isolated under sterile conditions. The brains were rinsed with cold PBS, sectioned into fragments, and digested with 0.25% trypsin at 37°C for 10 min. Digestion was halted using Dulbecco's Modified Eagle Medium (DMEM, Gibco, Cat# 11965092) supplemented with 10% fetal bovine serum (FBS, HyClone, Cat# SH30084.03HI). Single‐cell suspensions were generated by gentle pipetting, followed by centrifugation to collect the cells. Cells were resuspended in 10% FBS/DMEM and transferred to poly‐L‐ornithine‐coated T‐25 flasks for cultivation in a 37°C incubator with 5% CO2. The medium was completely replaced after 2 days, and half of the medium was replaced every 2 days for the next 3–8 days. After 10‐14 days, T‐25 flasks were shaken at 180 rpm for 1 h at 37°C to separate microglia from astrocytes and oligodendrocytes. The collected medium was centrifuged, and cell pellets were resuspended in fresh medium. Further, due to the limitation that in vitro cultured microglia are unable to sustain their homeostasis independently, to maintain the normal function of microglia, cells were diluted in TIC media [75, 76] (containing TGF‐β, IL‐34, and cholesterol) with 2% FBS before seeding 1 × 10^5^ cells per 500 µL in a well of poly‐L‐ornithine‐coated 12‐well or 24‐well plate. The cells were maintained in TIC media at 37°C and 5% CO2, with media being changed per day until LPS administration (about 1–2 days in vitro). The BV‐2 microglia cell line, provided by Naval Medical University (Shanghai, China), was cultured in DMEM with 10% FBS in a 37°C incubator with 5% CO2. Cells were stimulated with 500 ng/ml lipopolysaccharide (LPS, MedChemoExpress, Cat#HY‐D1056) or non‐stimulated with an equivalent volume of PBS for 24 h.

### 5.10 RNA‐seq

Total RNA was isolated using TRIzol reagent (Thermo Fisher Scientific, Cat# 15596018CN,), and mRNA was subsequently enriched with Oligo(dT) beads and fragmented into shorter pieces. The mRNA fragments were reverse‐transcribed into cDNA using random primers. The resulting cDNA fragments were purified with the QiaQuick PCR extraction kit, followed by end repair, poly(A) tailing, and ligation to Illumina sequencing adapters. The ligation products were size‐selected via agarose gel electrophoresis, PCR‐amplified, and sequenced on the Illumina HiSeq2500 platform. Raw reads were processed to filter out those containing adapters, more than 10% ambiguous nucleotides, or more than 50% low‐quality bases (Q‐value ≤ 20). Reads mapping to rRNA were removed using Bowtie2, and the remaining reads were mapped to the reference genome by TopHat2 (version 2.0.3.12). Gene abundance was quantified using RSEM software and normalized by the TPM (transcripts per million) method.

### 5.11 Western Blotting

Cells were washed with cold PBS and lysed using RIPA buffer containing protease and phosphatase inhibitors. The lysates were subjected to ultrasonic disruption, followed by centrifugation at 12 000 rpm for 15 min at 4°C. Protein concentrations were measured using a BCA protein assay kit. The lysates were then diluted with 5× SDS loading buffer and heated at 95°C for 10 min. Equal amounts of total protein were loaded onto 10% or 12.5% SDS‐polyacrylamide gels, separated by electrophoresis, and transferred onto PVDF membranes using the wet transfer method. Membranes were blocked with protein‐free rapid blocking buffer for 30 min at room temperature and subsequently incubated overnight at 4°C with primary antibodies (Table S1). After washing, membranes were incubated with HRP‐conjugated goat anti‐rabbit or anti‐mouse IgG secondary antibodies for 2 h at room temperature. Immunoreactive bands were detected using enhanced chemiluminescence reagents.

### 5.12 Quantitative Polymerase Chain Reaction (qPCR)

Total RNA was extracted from cultured cells using Trizol following the manufacturer's instructions. The RNA was quantified using a Nanodrop 2000 (Thermo Fisher Scientific) and reverse‐transcribed to cDNA using a reverse transcription kit. qPCR was performed using the SYBR Green kit on a Quant Studio 5 (Applied Biosystems). The cycle threshold (CT) values of target genes were normalized to β‐actin, and fold changes in gene expression were calculated using the 2^‐ΔΔCT^ method. Primer sequences are listed in Table S2.

### 5.13 Seahorse XF Cell Mito Stress Test

Oxygen consumption rates (OCR) were measured using an XF‐96 Extracellular Flux Analyzer (Agilent Technologies). OCR was measured under basal conditions and following the sequential addition of 1 µM oligomycin, 1 µM fluorocarbonyl cyanide phenylhydrazone, and 100 nM rotenone + 1 µM antimycin A. For fatty acid oxidation (FAO) ratio calculation, microglia were treated with 40 µM etomoxir (MedChemoExpress, Cat#HY‐50202) or DMSO (MedChemoExpress, Cat#HY‐Y0320) for 1 h before OCR measurement [40].

### 5.14 Immunofluorescence (IF) of Primary Microglia

Cells seeded on round coverslips in a 48‐well plate were fixed with PFA for 10 min, washed three times with PBS, and incubated with primary antibodies (Table S1) overnight at 4°C after blocking with 10% donkey serum and 0.2% Triton‐X‐100 for 1 h at room temperature. Cells were then incubated with fluorescence‐labeled secondary antibodies. A confocal microscopy (Cytation C10, BioTek) was used to visualize stained cells. Fluorescence intensity was measured using ImageJ.

### 5.15 Chromatin Immunoprecipitation (ChIP)

Chromatin immunoprecipitation was performed using the SimpleChIP Enzymatic Chromatin IP Kit (Cell Signaling Technology, #9003). Cells were incubated with 1% formaldehyde at room temperature for 10 min to cross‐link chromatin, with the reaction terminated by incubation with glycine for 5 min. Cell nuclei were isolated in cold buffer with DTT and protease inhibitor cocktail for 10 min, and chromatin was digested with micrococcal nuclease at 37°C for 20 min. Digested chromatin was released through ultrasonic sonication, and fragments were confirmed by agarose gel electrophoresis to be 150–900 bp. Primary antibodies against H3R2me2a and IgG (supplied in the kit) were used for ChIP. qPCR was performed to quantify the level of *Ppargc1a* promoter in precipitated DNA samples, with data presented as the percentage of total input DNA.

### 5.16 siRNA Construction and Transfection

siRNA targeting mouse PGC‐1α was constructed by OBIO Biotechnology, including two different siRNAs targeting PGC‐1α: siRNA1 (5′‐3′): CCGCAAUUCUCCCUUGUAUTT, siRNA2 (5′‐3′): CCCACAGGAUCAGAACAAATT and control siRNA (5′‐3′): UUCUCCGAACGUGUCACGUTT. Primary microglia were cultured in 24‐well plates at a density of 2 × 10^4^ cells/cm^2^. Cells were transfected with siRNA targeting PGC‐1α or control siRNA for 6 h using the transfection reagent Lipofectamine 2000 diluted in Opti‐MEM. The medium was then replaced with 10% FBS/DMEM, and cells were cultured for an additional 48 h. Western blotting, qPCR, and immunofluorescence assays were used to evaluate siRNA efficiency.

### 5.17 Plasmid Construction and Transfection

Previously constructed plasmids, including H3.3, H3.3R2A. And the scrambling sequence was used as a control [26]. BV‐2 cells were cultured in 24‐well plates until they reached 50%–60% density. Plasmid transfection in BV‐2 cells was performed using jetPEI‐Macrophage DNA Transfection Reagent (polyplus, #103‐05N), following the manufacturer's instructions. Briefly, plasmids and jetPEI‐Macrophage were diluted into 150 mM NaCl respectively, followed by mixture. Cells were incubated with the mixed solution for 6 h and then the medium was changed with DMEM containing 10% FBS. Following transfection, BV‐2 cells were treated with 10 µM EPZ020411 or an equivalent volume of DMSO (control) for 24 h before 24 h LPS/PBS treatment.

### 5.18 Statistical Analysis

All data were analyzed using GraphPad Prism v9.3.0 (GraphPad Software) and are presented as mean ± standard error of the mean (SEM). Unpaired t‐test was used to compare differences between two groups. One‐way ANOVA was employed to compare differences among three or more groups, followed by Tukey's multiple comparisons test. A *p*‐value < 0.05 is considered statistically significant.

## Author Contributions

W.P., Z.W., and Y.X. contributed equally to this work. X.L. and C.H. conceptualized and designed the study. W.P., W.C., and Y.X. carried out experiments and analyzed data. Z.W. analyzed bioinformation. Y.X. and Y.L. made the figures and tables. Z.G. and Z.W. drafted the paper. H.W. revised the paper. All authors discussed and commented on the manuscript.

## Conflicts of Interest

The authors declare no conflict of interests.

## Supporting information




**Supporting File 1**: advs75325‐sup‐0001‐SuppMat.docx.

**Supporting File 2**: advs75325‐sup‐0002‐Data.zip.

## Data Availability

The data that support the findings of this study are available from the corresponding author upon reasonable request.
