# Supplementary material for: Prmt6 Deficiency or Inhibition Restores Microglial Homeostasis and Promotes Scar‐Limited Repair in Adult Spinal Cord Injury
Source: Adv Sci (Weinh). 2026 Apr 17;13(38):e75325. doi: 10.1002/advs.75325 (PMC13335747; doi:10.1002/advs.75325)
Supplement: Supplementary file 1 — Supporting File 1: advs75325‐sup‐0001‐SuppMat.docx. [file ADVS-13-e75325-s001.docx]

Supporting Information

Prmt6 deficiency or inhibition restores microglial homeostasis and promotes scar-limited repair in adult spinal cord injury

Weilin Peng, Zhengqiang Wu, Yu Xiong, Zhongya Gao, Yishan Liu, Ziyi Wang, Haibin Wang, Chaofeng Han *, Wenxiang Chu*, and Xuhua Lu *


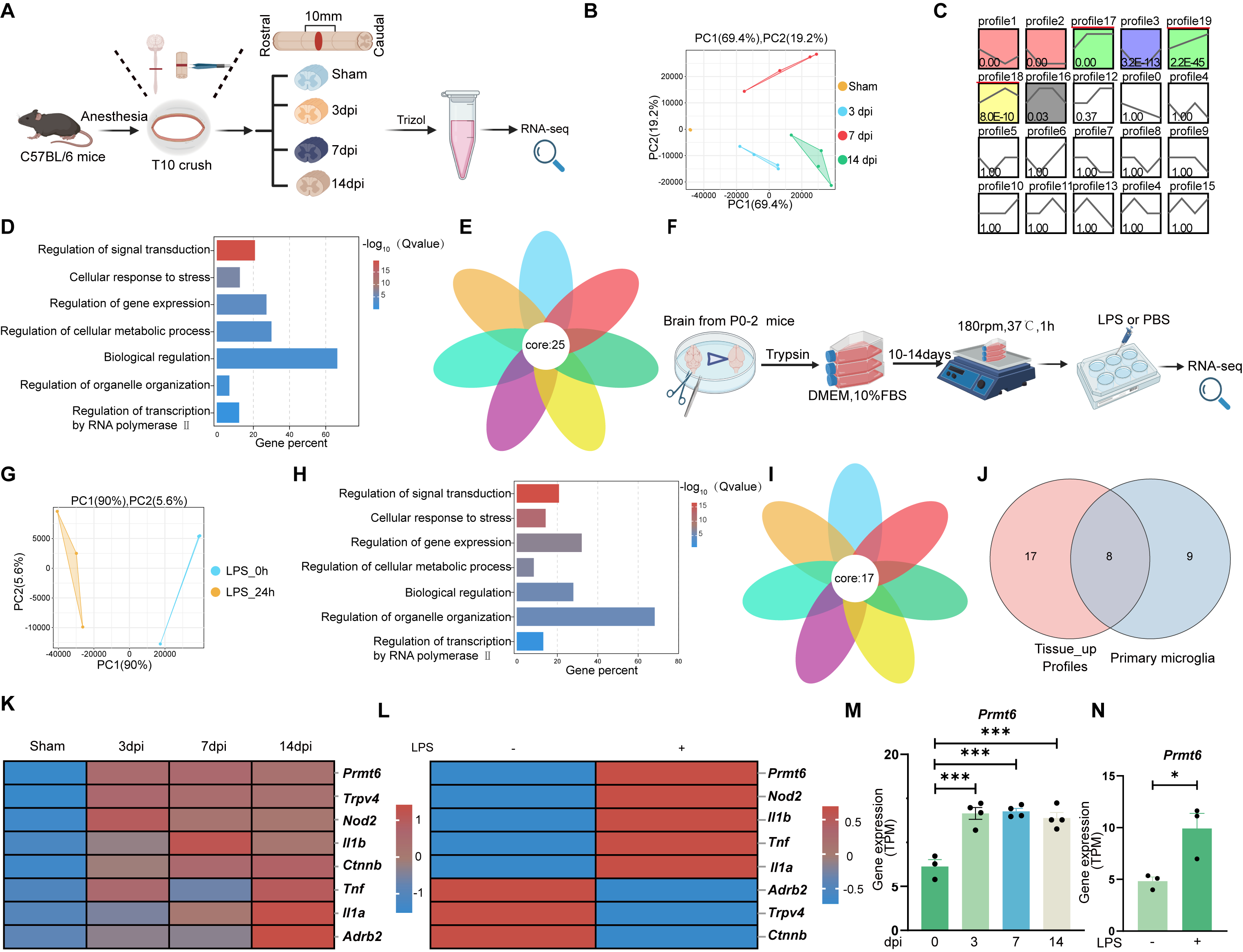


Related to Figure 1

**Figure S1.** Identification of *Prmt6* upregulation after adult SCI through RNA-seq. A) Schematic illustration of spinal cord crush injury and RNA-seq. Created in BioRender. Weilin, Peng. (2026) [https://BioRender.com/tl2mwmh.](https://BioRender.com/tl2mwmh) B) Principal component analysis (PCA) between spinal cords harvested from uninjured and injured mice at 3, 7 and 14 dpi. C) Trend analysis of uninjured and injured spinal cords harvested at 3, 7, and 14 dpi. Profiles containing persistently upregulated genes (profile 17, 18, and 19) were highlighted. p-values are shown in the corner. D) Gene Ontology (GO) analysis of gene profiles (profile 17, 18, and 19 in C) containing persistently upregulated genes post-SCI. E) Venn diagram analysis illustrating the overlap among GO terms from D, identifying 25 candidate genes. F) Schematic representation of primary microglia culture and RNA-seq. Created in BioRender. Weilin, Peng. (2026) [https://BioRender.com/tl2mwmh.](https://BioRender.com/tl2mwmh) G) Principal component analysis (PCA) of primary microglia treated or untreated with LPS. H) GO enrichment analysis comparing activated and non-activated primary microglia, highlighting identical GO terms to those identified *in vivo*. I) Venn diagram displaying the 17 candidate genes identified at the intersection of GO terms from F. J) Venn diagram illustrating the intersection of candidate genes identified from spinal cord tissue. K) Heatmaps of core genes expression in RNA-seq data from spinal cord and L) heatmaps of core genes expression in RNA-seq data from primary microglia. M) Upregulated expression of *Prmt6* from RNA-seq data *in vivo*. N) Upregulated expression of *Prmt6* from RNA-seq data of primary microglia.


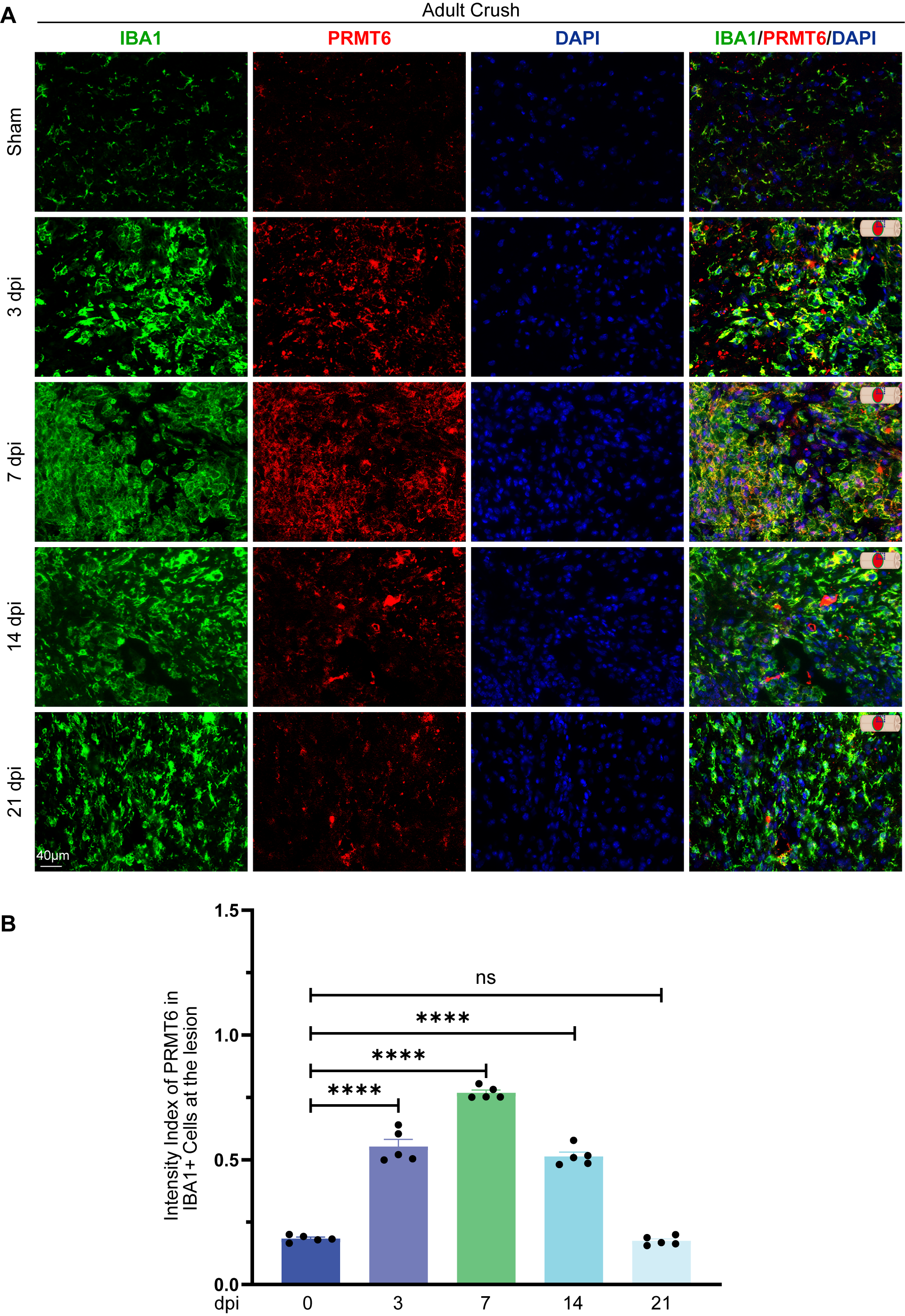


Related to Figure 1

**Figure S2.** PRMT6 upregulation in microglia after spinal cord injury. A, B) Co-staining of PRMT6 and IBA1 (A) and quantification of PRMT6 expression in IBA1^+^ cells (B) in intact spinal cord and spinal cord lesion at 3, 7, 14, and 21 dpi, confirming PRMT6 upregulation after SCI in 3, 7, and 14 dpi. Values are plotted as means ± SEM. ****P < 0.0001.


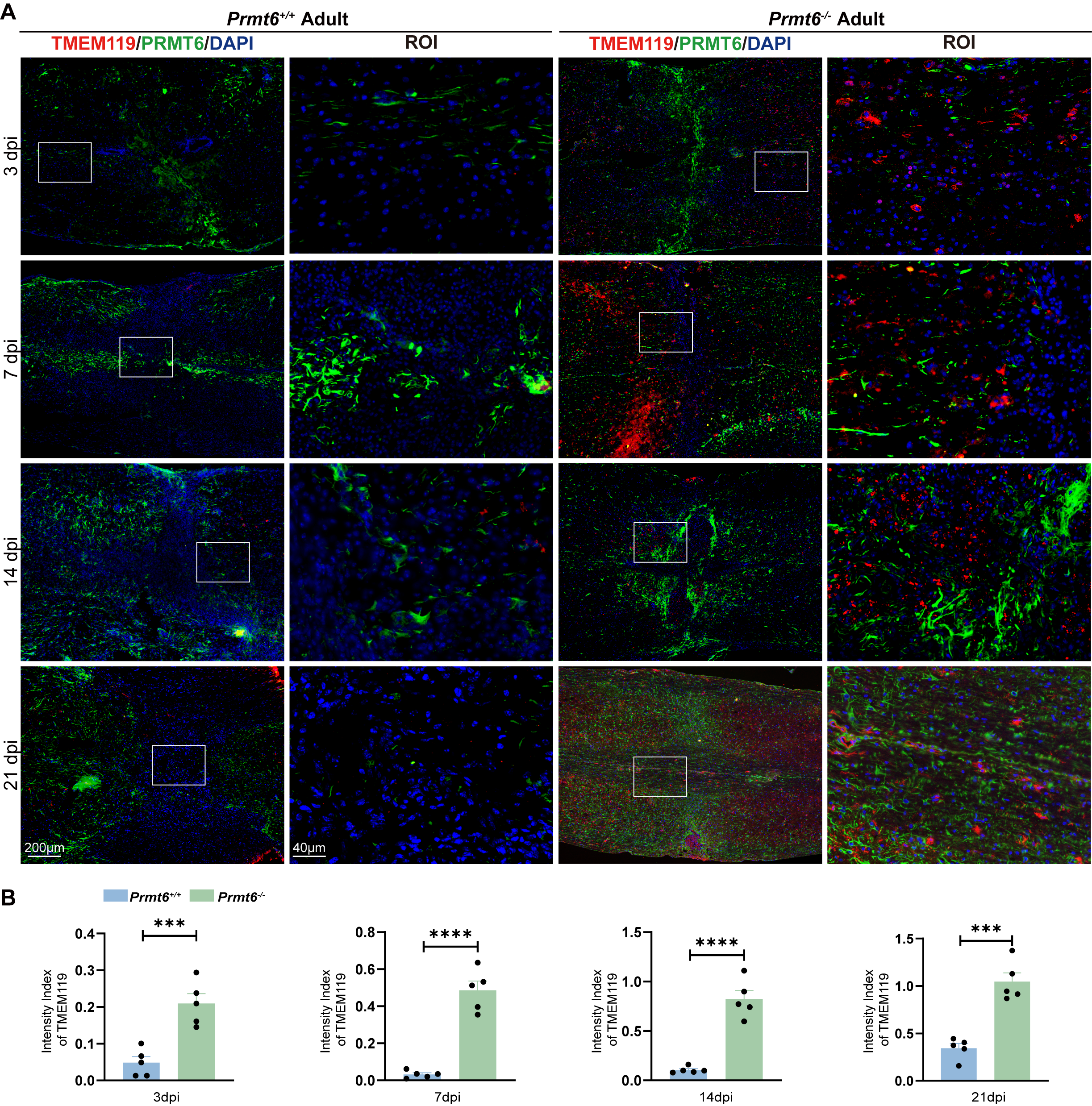


Related to Figure 2

**Figure S3.** Microglia in *Prmt6*^-/-^ mice re-expressed TMEM119 after SCI. A) Representative images of spinal cord lesions of *Prmt6*^+/+^ and *Prmt6*^-/-^ mice at different time points after SCI, stained with antibodies against TMEM119 (red) and GFAP (green), and nuclei were stained with DAPI (blue). White square indicates the region of interest (ROI). B) Quantification of TMEM119 immunoreactive intensity (normalized to proximal intact region) in the lesion sites of *Prmt6*^+/+^ and *Prmt6*^-/-^ mice at 3, 7, 14, and 21days post-injury. Values are plotted as means ± SEM. ***P < 0.001, ****P < 0.0001.


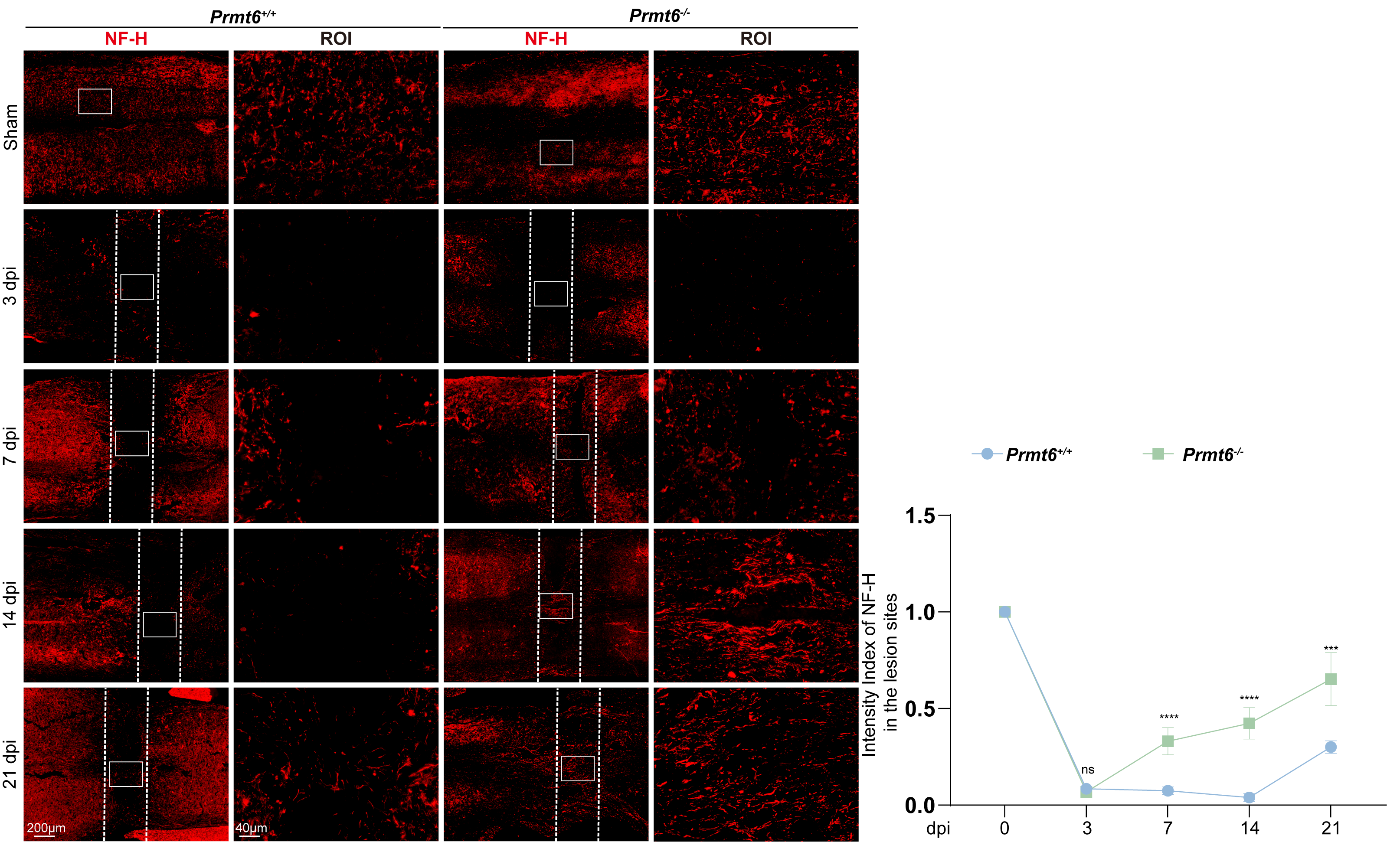


Related to Figure 3

**Figure S4.** Promoted axonal regrowth in *Prmt6*-deficiency mice. Representative images and quantification of NF-H^+^ axons in lesions of *Prmt6*^+/+^ and *Prmt6*^-/-^ mice post-SCI, suggesting increased NF-H+ axons intensity in *Prmt6*^-/-^ mice compared to their *Prmt6*^+/+^ counterparts at the lesion area at 7, 14, and 21 dpi. White square indicates the region of interest (ROI), and dotted lines indicate the lesion sites. Intensity of NF-H within the dotted area was quantitated. Values are plotted as means ± SEM. ***P < 0.001, ****P < 0.0001.


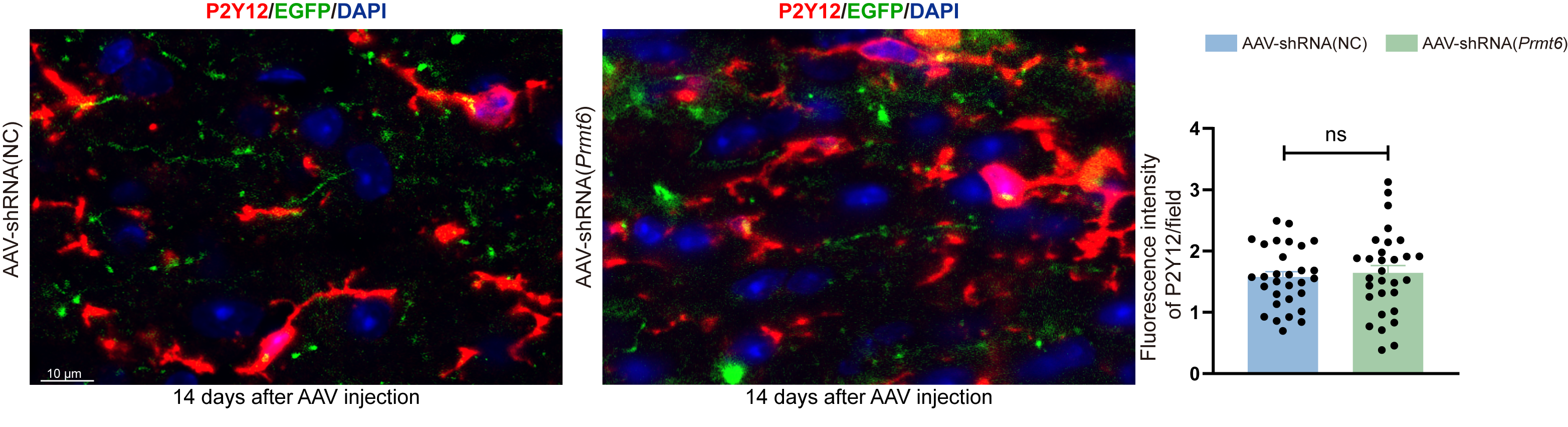
Related to Figure 4

**Figure S5.** AAV-Cx3cr1-EGFP-miR30shRNA (*Prmt6*) and AAV-Cx3cr1-EGFP-miR30shRNA (NC) did not disrupt microglial homeostasis in intact spinal cord. Images of immunostaining for P2Y12 and EGFP and quantitative analysis for P2Y12 in spinal cord infected with AAV-Cx3cr1-EGFP-miR30shRNA (*Prmt6*) or AAV-Cx3cr1-EGFP-miR30shRNA (NC), respectively (n = 10 cells/animal, 3 animals per group). Values are plotted as means ± SEM.


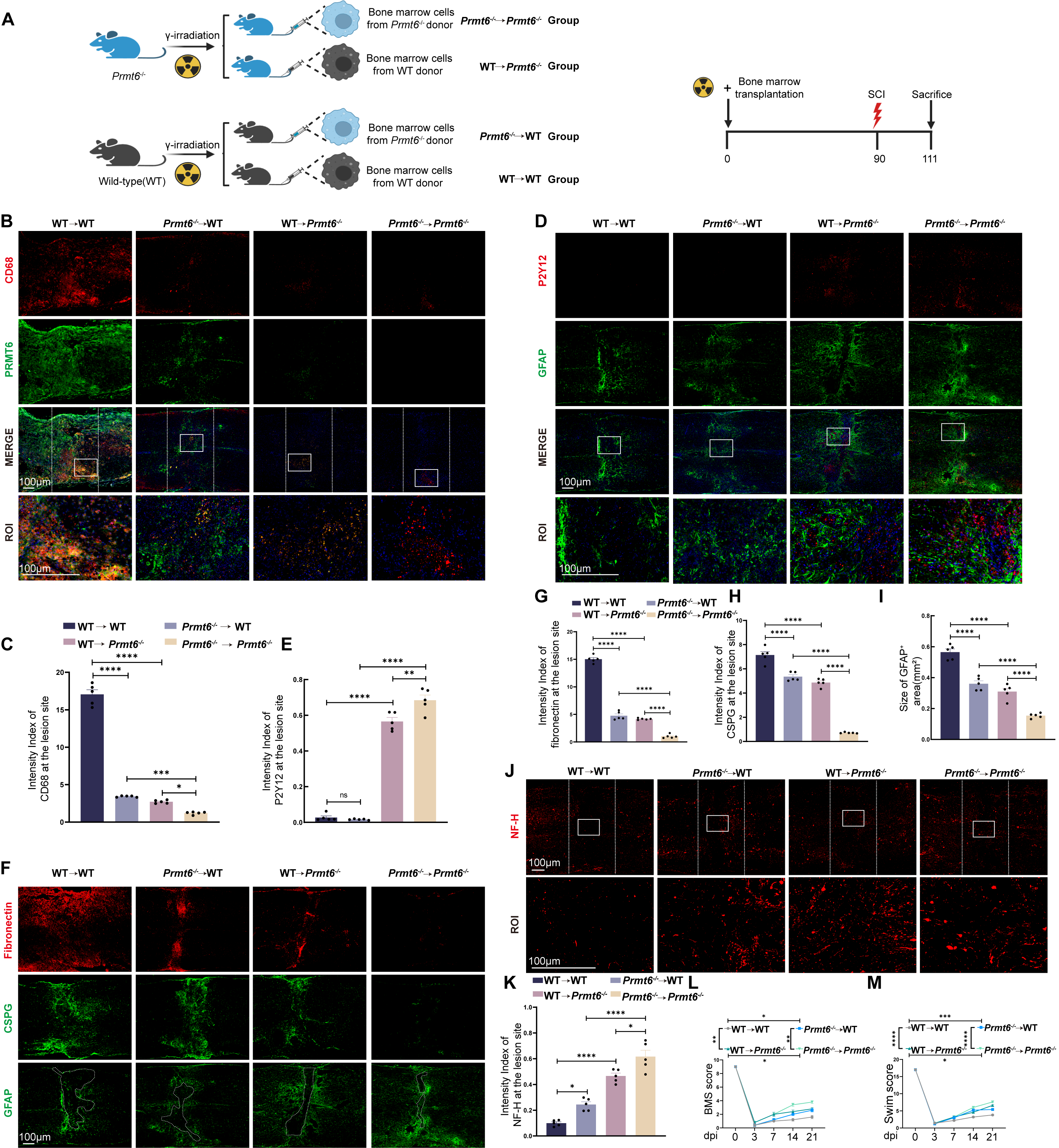
Related to Figure 4

**Figure S6.** *Prmt6* deficiency in blood-borne cells was not necessary for the restoration of microglial homeostasis. A) Schematic illustration showing the generation of chimeric mice and the experimental strategy. Created in BioRender. Weilin, Peng. (2026) [https://BioRender.com/tl2mwmh.](https://BioRender.com/tl2mwmh) B) Co-staining of CD68 and PRMT6 and C) quantification of CD68 intensity in spinal cord sections from different groups at 21 dpi (n = 5 per group). D, E) Co-staining of P2Y12 and GFAP (D) and quantification of P2Y12 intensity (E) in spinal cord sections from different groups at 21 dpi (n = 5 per group). F) Representative images of spinal cord sections from different groups at 21 dpi, stained with antibodies against fibronectin (red), GFAP (green), or CSPG (green), which suggests reduced scar formation in WT mice transplanted with *Prmt6*-deficient bone marrow. To note, the images of GFAP staining was obtained from (D). G, H) Quantification of fibronectin (G) and CSPG (H) intensity in the lesion sites at 21 dpi. I) Quantification of the size of lesion area bordered by GFAP at 21 dpi. J, K) Immunofluorescence(J) and quantitative analysis(K) of NF-H in spinal cord sections from different groups at 21 dpi. White square indicates the ROI, and dotted lines indicate the lesion sites. L, M) Statistical analysis of the BMS (L) and Louisville Swim Scale (M) for different groups over a 21-day period. Statistical analysis was performed on the scores measured at day 21. Values are plotted as means ± SEM. *P < 0.05, **P < 0.01, ***P < 0.001, ****P < 0.0001.


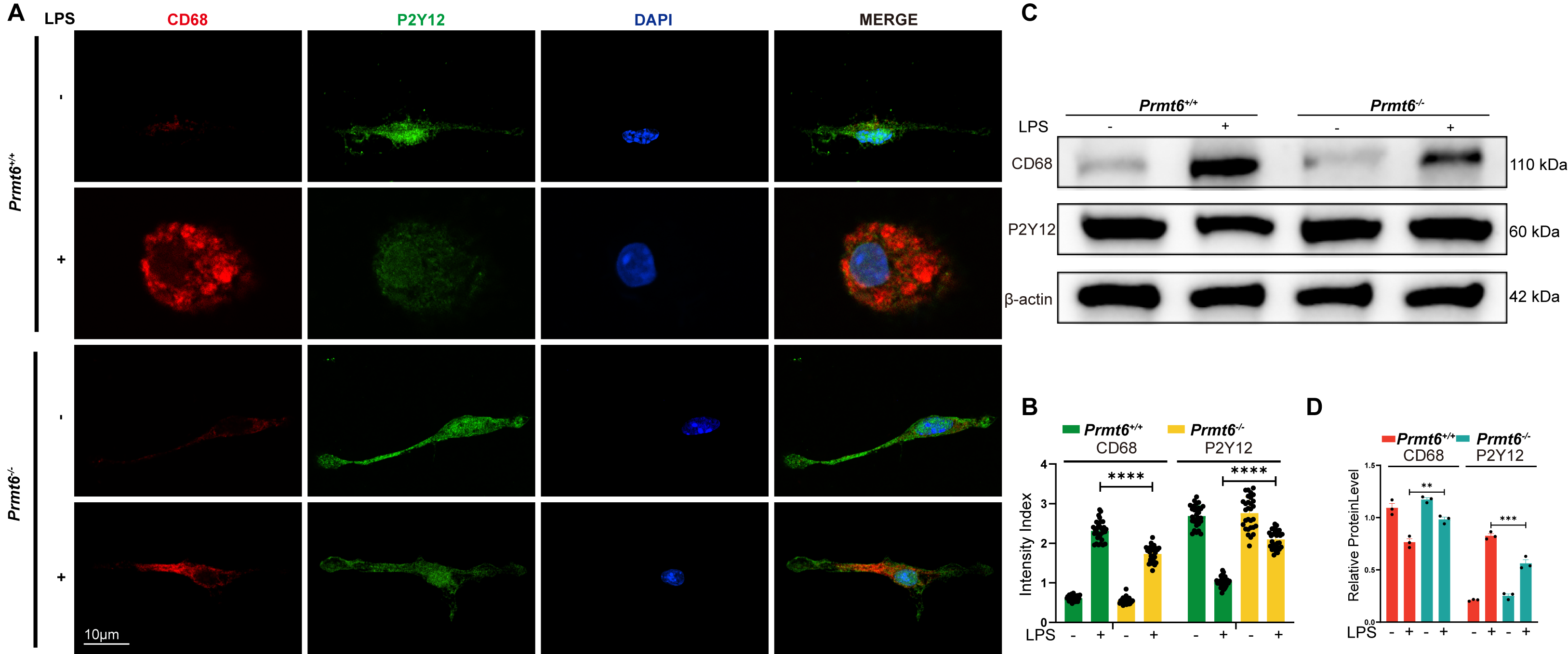


Related to Figure 6

**Figure S7.** The LPS-activated primary microglia model recapitulated *Prmt6*-dependent regulation of microglial homeostasis in vitro. A, B) Representative immunofluorescence images (A) and quantification (B) of CD68 (red) and P2Y12 (green) in *Prmt6*^+/+^ and *Prmt6*^-/-^ primary microglia after 24 h LPS stimulation, showing higher P2Y12 and lower CD68 expression in *Prmt6*^-/-^ cells. C, D). Western blots (C) and quantification (D) of CD68 and P2Y12 expression in *Prmt6*^+/+^ and *Prmt6*^-/-^ primary microglia before and after LPS treatment, confirming that *Prmt6* deficiency preserves a more homeostatic marker profile upon activation. Values are plotted as means ± SEM. **P < 0.01, ***P < 0.001, ****P < 0.0001.

.


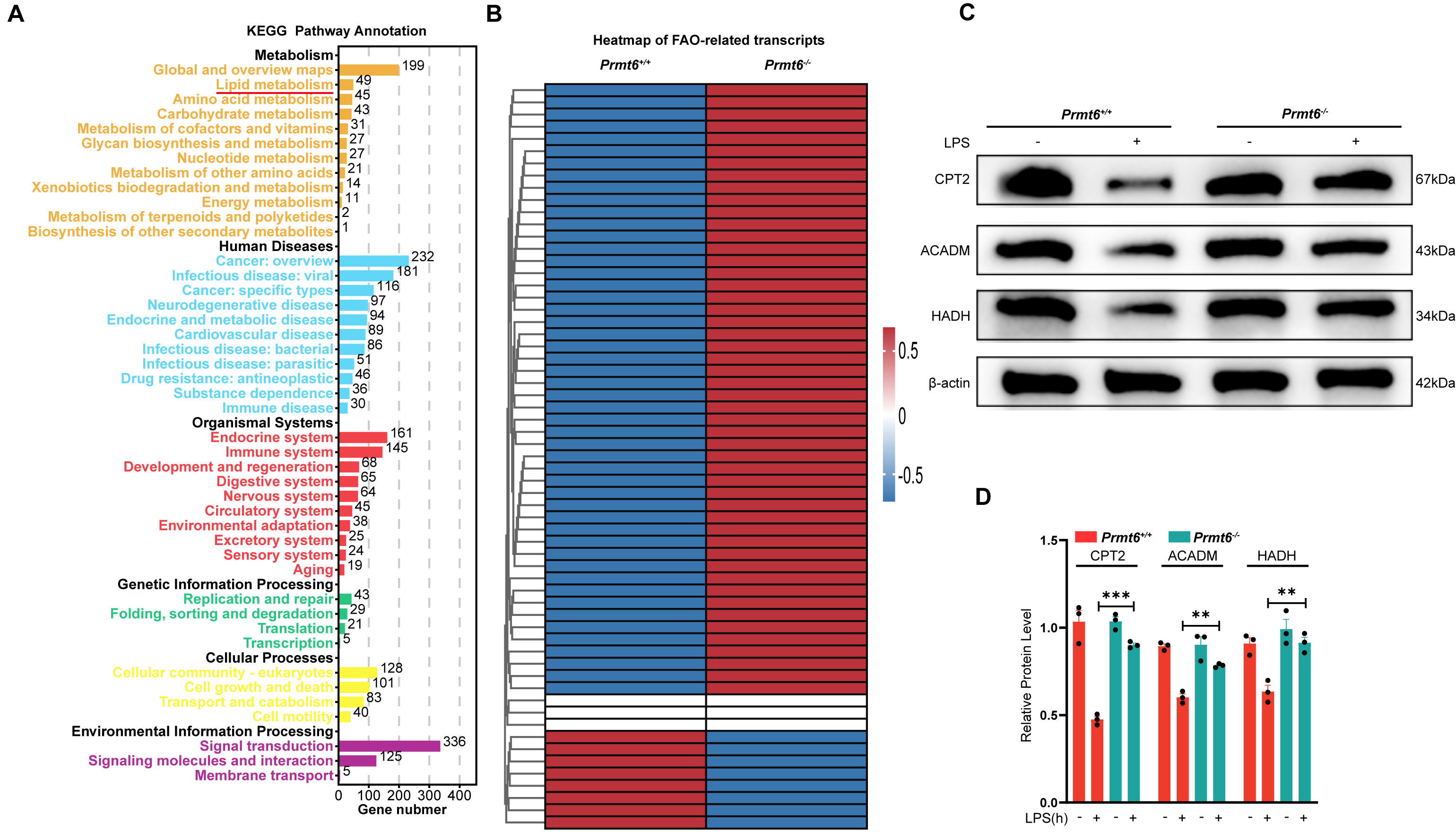
Related to Figure 6

**Figure S8.** *Prmt6*^-/-^ primary microglia exhibited enhanced fatty acid oxidation (FAO) after LPS stimulation compared to *Prmt6*^+/+^ cells. A) KEGG term analysis shows differed pathways between *Prmt6*^+/+^ and *Prmt6*^-/-^ primary microglia after LPS activation B) Heatmap analysis shows *Prmt6* deficiency restores expression of FAO-related genes in primary microglia after LPS stimulation. C, D) Western blots (C) and quantitative analysis (D) demonstrate increased expression of FAO-related enzymes (CPT2, ACADM, and HADH) in *Prmt6*^-/-^ primary microglia compared to *Prmt6*^+/+^ cells. Values are plotted as means ± SEM. **P < 0.01, ***P < 0.001, ****P < 0.0001.


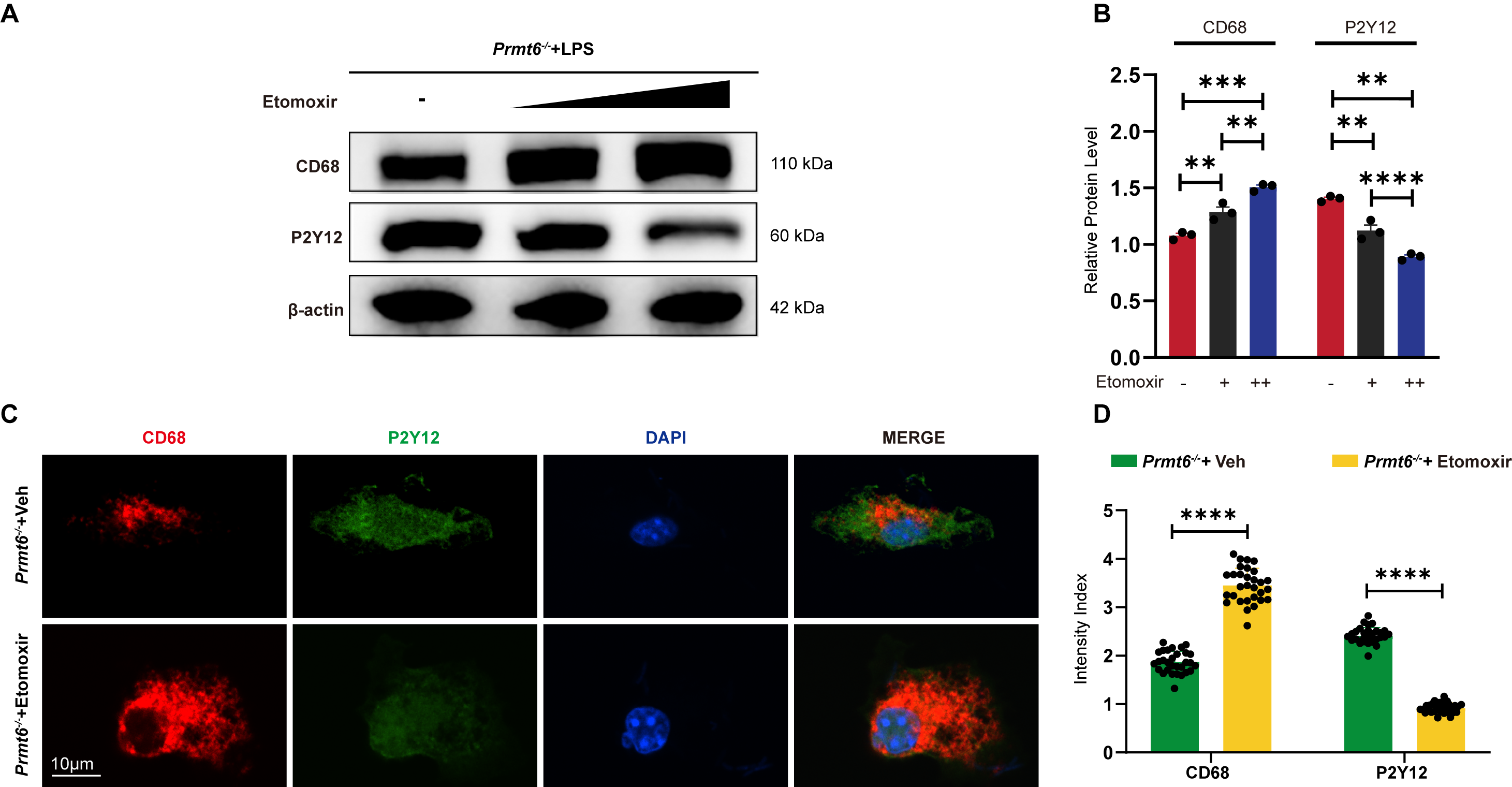


Related to Figure 6

**Figure S9.** Inhibiting FAO abolished the homeostasis maintained by *Prmt6* deficiency in primary microglia. A, B) Western blot analysis (A) and quantitative analysis (B) of the activation marker CD68 and the homeostatic marker P2Y12 in LPS-treated *Prmt6*^-/-^ microglia treated with a concentration gradient of etomoxir. C, D) Representative confocal images (C) and quantitative analysis (D) of LPS-treated *Prmt6*^-/-^ microglia stained with CD68(red) and P2Y12(green) with or without etomoxir treatment. Values are plotted as means ± SEM. **P < 0.01, ***P < 0.001, ****P < 0.0001.


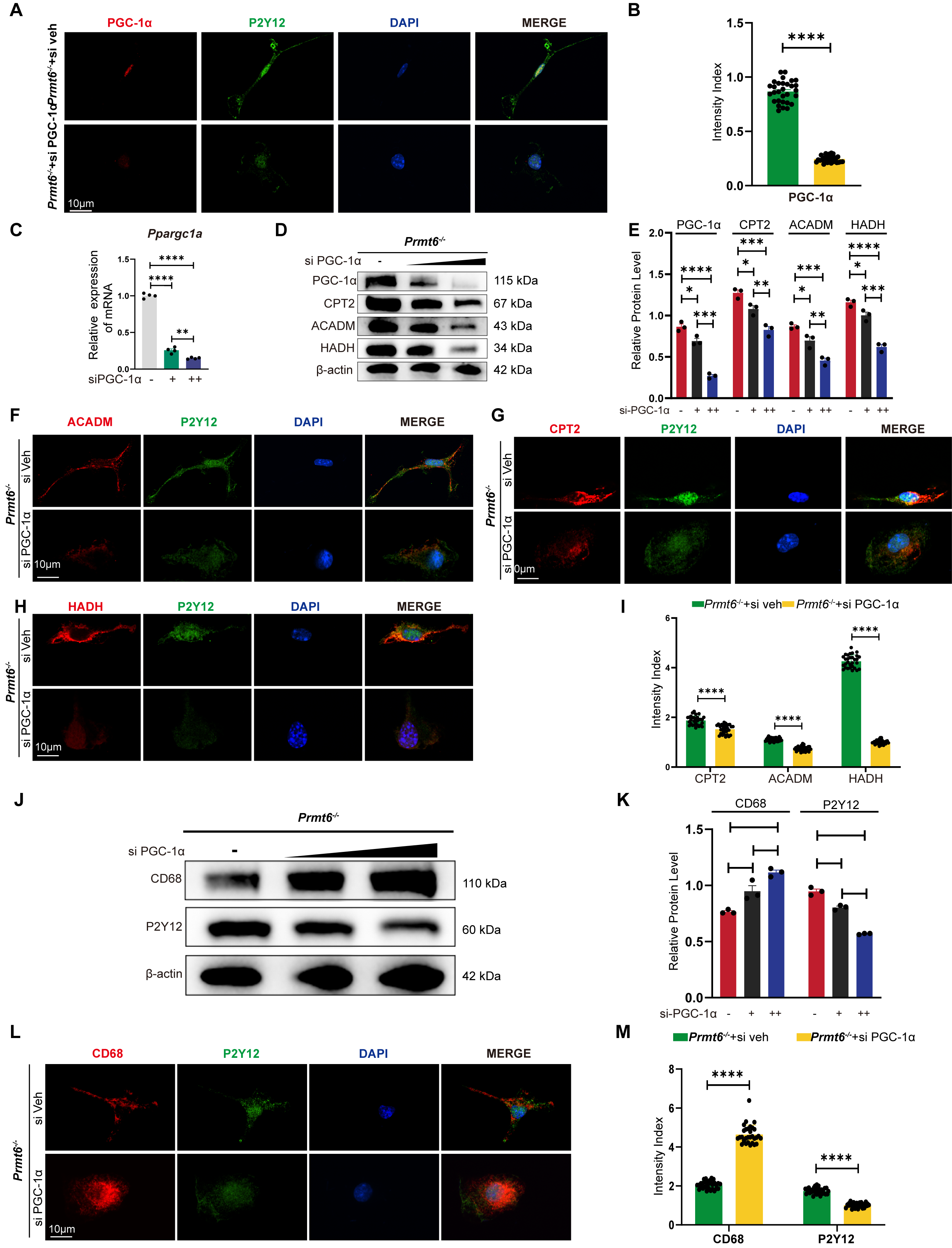


Related to Figure 7

**Figure S10.** Transfection of siPGC-1α downregulated PGC-1α-mediated FAO in *Prmt6*^-/-^ microglia after LPS treatment. A, B) Representative immunofluorescence images (A) of PGC-1α (red) co-stained with P2Y12(green) and quantitative analysis (B) identify the downregulated fluorescence intensity of PGC-1α in LPS-treated *Prmt6*^-/-^ microglia transfected with siPGC-1α or control siRNA. C) Quantitative PCR (qPCR) analysis of Ppargc1a mRNA levels in LPS-activated *Prmt6*^-/-^ microglia treated with or without PGC-1α siRNA, indicating a dose-dependent downregulation. D, E) Western blot analysis (D) and quantification (E) of relative protein levels of PGC-1α and its downstream targets, including CPT2, ACADM, and HADH, in *Prmt6*^-/-^ microglia treated with or without PGC-1α siRNA. F-I) Representative confocal images of CPT2 (F), ACADM (G), and HADH (H) (red) co-stained with P2Y12 (green) and quantitative analysis (I) in LPS-activated *Prmt6*^-/-^ microglia transferred with PGC-1α siRNA or control siRNA. J, K) Western blot analysis (J) and quantification (K) of the activation marker CD68 and the homeostatic marker P2Y12 in *Prmt6*^-/-^ microglia transfected with PGC-1α siRNA or control. L, M) Representative immunofluorescence images (L) and quantitative analysis (M) of CD68 and P2Y12 in *Prmt6*^-/-^ microglia transfected with siRNA-PGC-1α or control (n = 30 per group). Values are plotted as means ± SEM. *P < 0.05, **P < 0.01, ***P < 0.001, ****P < 0.0001.


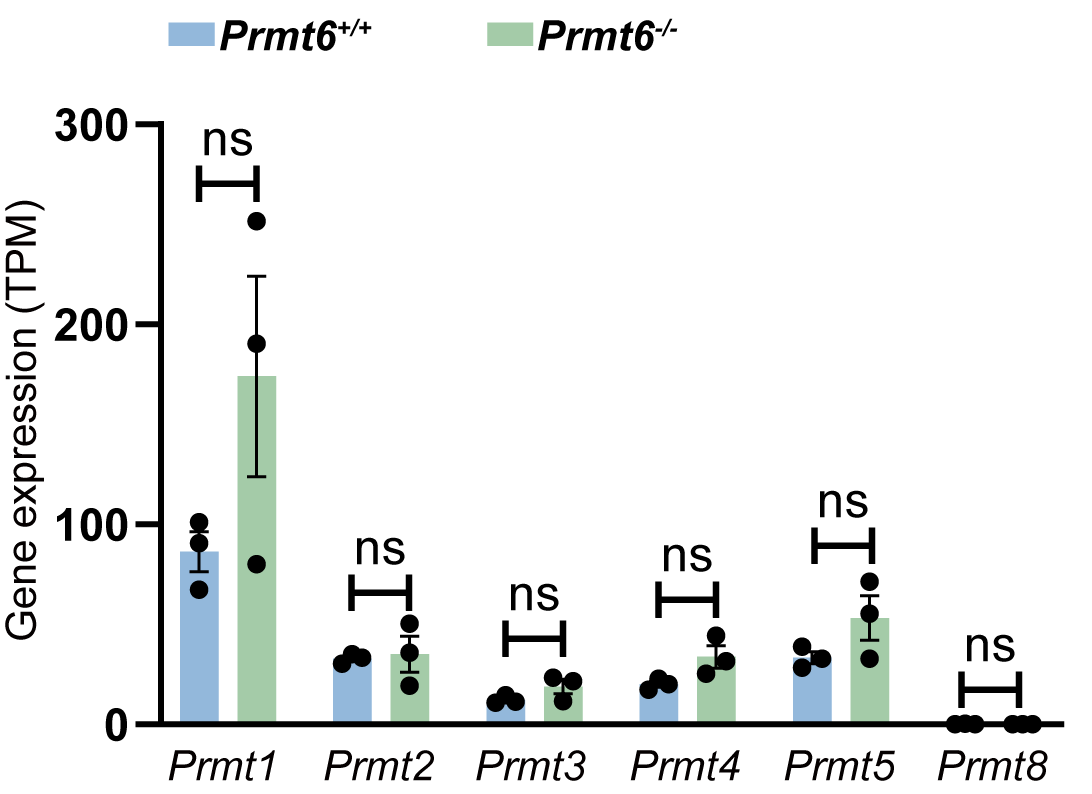


**Figure S11.** *Prmt6* deficiency did not disturb other PRMT expression in microglia. Expression of *Prmt1*, *Prmt2, Prmt3, Prmt4, Prmt5, Prmt8* (TPM) from RNA-seq data in primary microglia.


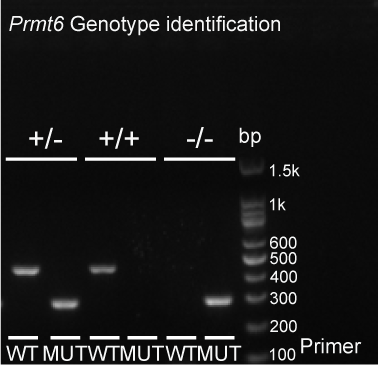


**Figure S12.** Genotype identification via PCR amplification. Identification of mouse genotypes was implemented through amplification of DNA samples using separate wild type (WT) and mutant (MUT) primers. As the resulting gel electrophoresis shows, *Prmt6*^+/+^ mice exhibited a distinct DNA band in the WT primer amplification lane, *Prmt6*^-/-^ mice displayed a DNA band only in the MUT primer amplification lane, while *Prmt6*^+/-^ mice exhibited bands in both lanes.

Table S1. Antibodies

| Antibodies | Source | Identifier | Dilution |
| --- | --- | --- | --- |
| Mouse monoclonal anti-PRMT6 antibody | Santa Cruz | Cat# sc-271744 RRID: AB_10715087 | 1: 200 |
| Rabbit polyclonal anti-PRMT6 antibody | Novus | Cat# NBP2-32459; RRID: AB_3282404 | 1: 500 |
| Rat monoclonal anti-CD68 antibody | Bio-Rad | Cat# MCA1957; RRID: AB_322219 | 1: 500 |
| Rabbit recombinant monoclonal anti-CD68 antibody | Abcam | Cat# ab283654; RRID: AB_2922954 | 1: 1000 |
| Rabbit monoclonal anti-P2Y12 antibody | Abcam | Cat# ab300140 | 1: 500 |
| Rabbit recombinant monoclonal anti-P2Y12 antibody | Abcam | Cat# ab184411; RRID: AB_2877713 | 1: 2000 |
| Rabbit monoclonal anti-TMEM119 antibody | Abcam | Cat# ab209064; RRID: AB_2800343 | 1: 500 |
| Mouse monoclonal anti-GFAP antibody | Cell Signaling Technology | Cat# 3670; RRID: AB_561049 | 1: 500 |
| Mouse monoclonal anti-NeuN antibody | Abcam | Cat#ab104224; RRID: AB_10711040 | 1：200 |
| Rabbit polyclonal anti-fibronectin antibody | Millipore | Cat# AB2033; RRID: AB_2105702 | 1：200 |
| Rabbit polyclonal anti-collagen I antibody | Abcam | Cat# ab21286; RRID: AB_446161 | 1: 200 |
| Mouse monoclonal anti-CSPG antibody | Sigma-Aldrich | Cat# C8035; RRID: AB_476879 | 1：200 |
| Rabbit monoclonal anti-NF-H antibody | Abcam | Cat# ab314077 | 1:200 |
| Rabbit recombinant monoclonal anti-MAP2 antibody | Abcam | Cat# ab254264; RRID: AB_2927400 | 1:500) |
| Rabbit polyclonal anti-PGC-1α antibody | Abcam | Cat# ab191838; RRID: AB_2721267 | 1:500 |
| Rabbit polyclonal anti-CPT2 antibody | Abcam | Cat# 181114; RRID: AB_2687503 | 1:1000 |
| Rabbit monoclonal anti-ADADM antibody | Abcam | Cat# ab92461; RRID: AB_10563530 | 1:500 |
| Rabbit polyclonal anti-HADH antibody | Proteintech | Cat#19828-1-AP; RRID: AB_10667408 | 1:2000 |
| Mouse monoclonal anti-β-Actin antibody | Cell Signaling Technology | Cat# 3700; RRID: AB_2242334 | 1:1000 |
| Rabbit recombinant monoclonal anti-H3 antibody | Cell Signaling Technology | Cat# 4499; RRID: AB_10544537 | 1:2000 |
| Rabbit polyclonal anti-H3R2me2a antibody | Abcam | Cat# ab175007 | 1:1000 |
| Goat anti-Rabbit IgG Alexa Fluor 594 antibody | Thermo Fisher | Cat# A-11012; RRID: AB_2534079 | 1:1000 |
| Goat anti- Rabbit IgG Alexa Fluor 488 antibody | Thermo Fisher | Cat# A-11008; RRID: AB_143165 | 1:500 |
| Goat anti-Mouse IgG Alexa Fluor 488 antibody | Thermo Fisher | Cat# A-11001; RRID: AB_2534069 | 1:1000 |
| Goat anti-Rat IgG Alexa Fluor 594 antibody | Thermo Fisher | Cat# A-11007; RRID: AB_10561522 | 1：500 |

Table S2. Primers

| Genes | Forward (5’- 3’) | Reverse (5’- 3’) |
| --- | --- | --- |
| β-actin | ACAGCAGTTGGTTGGAGCAA | ACGCGACCATCCTCCTCTTA |
| Ppargc1a | TATGGAGTGACATAGAGTGTGCT | CCACTTCAATCCACCCAGAAAG |
| Acadm | AGGGTTTAGTTTTGAGTTGACGG | CCCCGCTTTTGTCATATTCCG |
| Hadh | TCAAGCATGTGACCGTCATCG | TGGATTTTGCCAGGATGTCTTC |
| Cpt2 | CAGCACAGCATCGTACCCA | TCCCAATGCCGTTCTCAAAAT |
