# Supplementary figures and images for: Prmt6 Deficiency or Inhibition Restores Microglial Homeostasis and Promotes Scar‐Limited Repair in Adult Spinal Cord Injury
Source: Adv Sci (Weinh). 2026 Apr 17;13(38):e75325. doi: 10.1002/advs.75325 (PMC13335747; doi:10.1002/advs.75325)

# Figure 6K

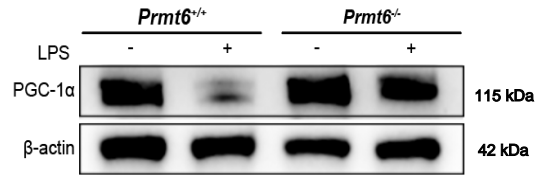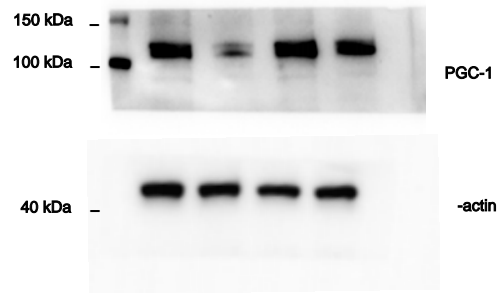

# Figure 8A

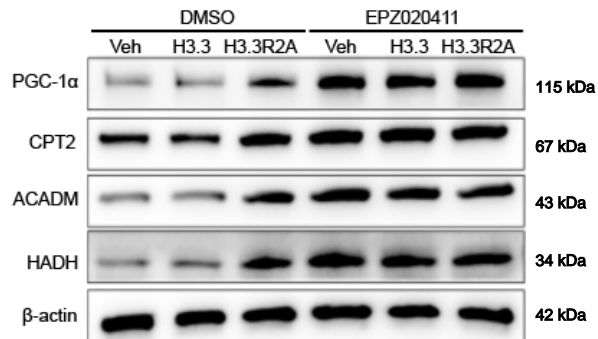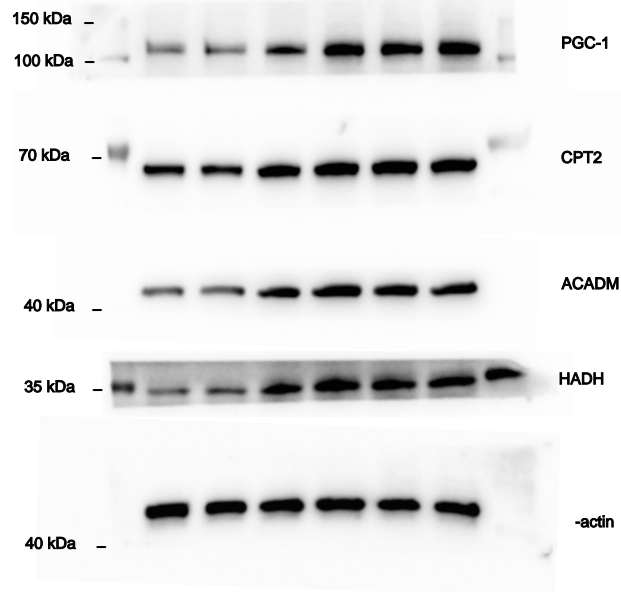

# Figure 8C

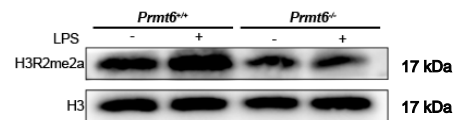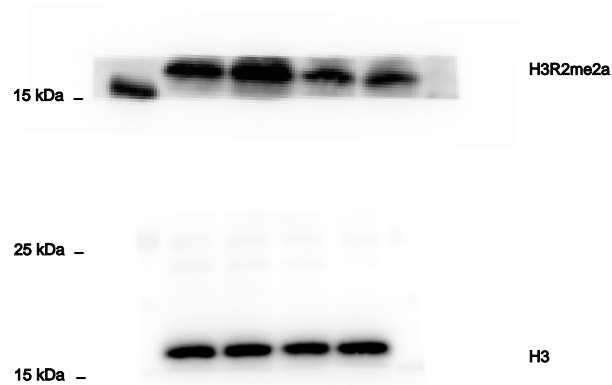

# Figure 8F

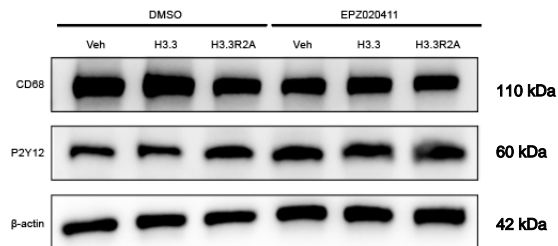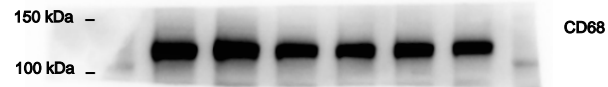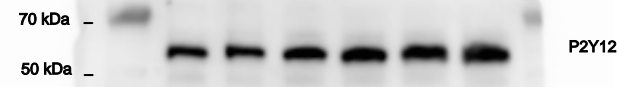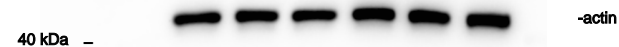

# Figure S7C

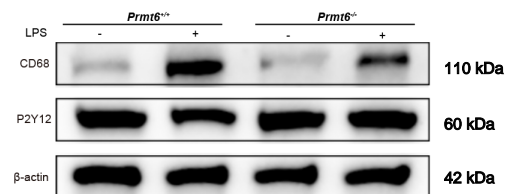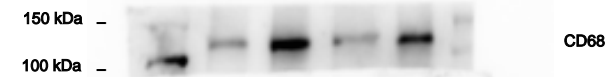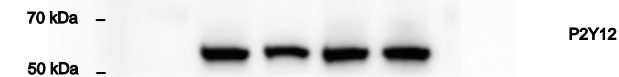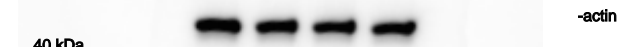

Figure S8C

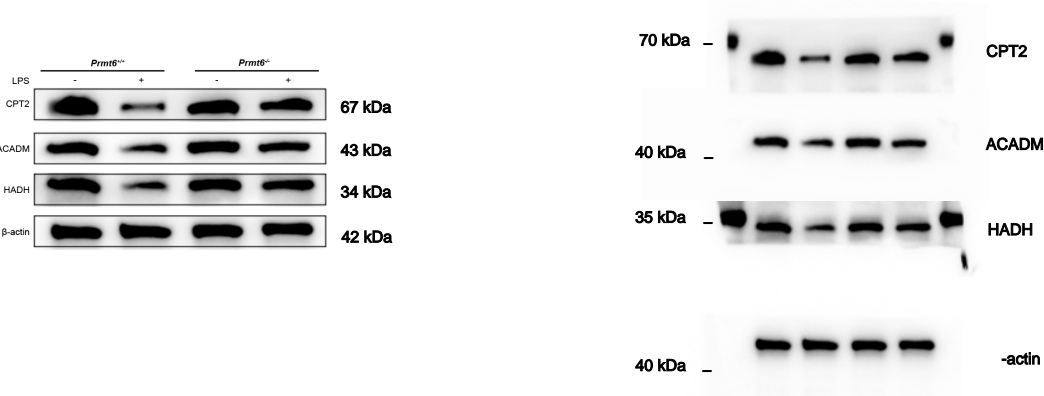

Figure S9A

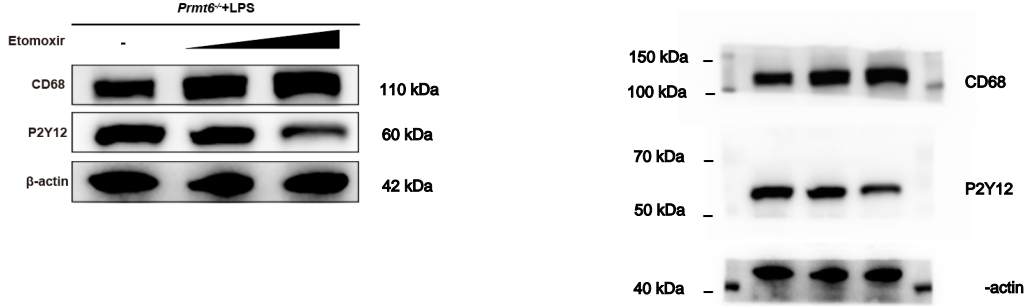

# Figure S10D

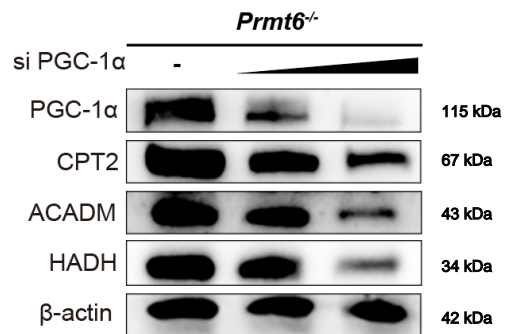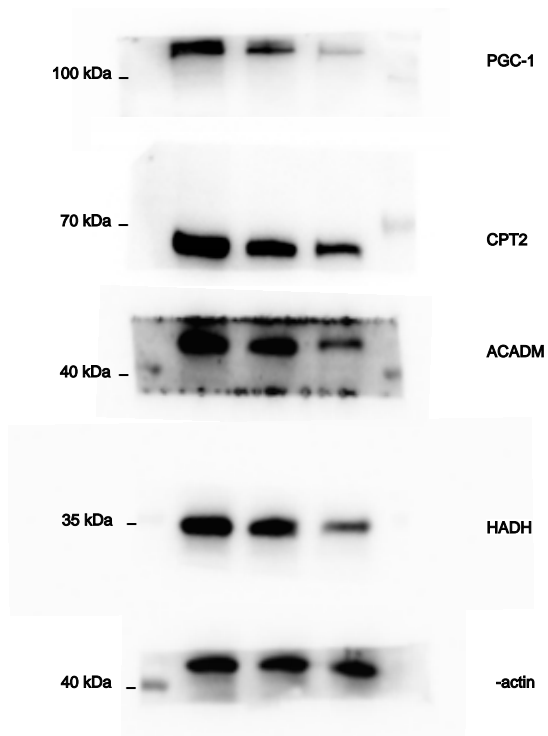

# Figure S10J

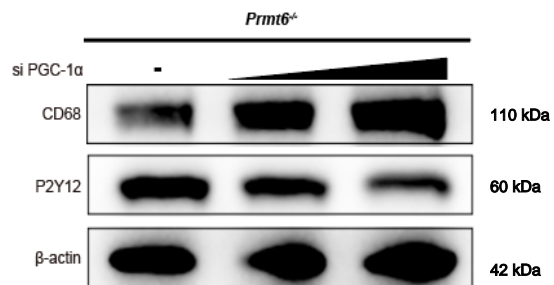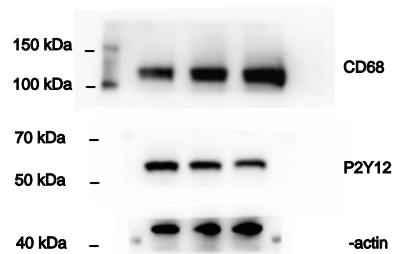

Supplement: Supplementary file 2 — Supporting File 2: advs75325‐sup‐0002‐Data.zip. [file ADVS-13-e75325-s002.zip › Supplementary Information_uncropped blots.pdf]
